# Evaluating the Robustness of Biomarkers of Dairy Food Intake in a Free-Living Population Using Single- and Multi-Marker Approaches

**DOI:** 10.3390/metabo11060395

**Published:** 2021-06-17

**Authors:** Katherine J. Li, Kathryn J. Burton-Pimentel, Elske M. Brouwer-Brolsma, Edith J. M. Feskens, Carola Blaser, René Badertscher, Reto Portmann, Guy Vergères

**Affiliations:** 1 Division of Human Nutrition and Health, Department of Agrotechnology and Food Sciences, Wageningen University & Research, P.O. Box 17, 6700 AA Wageningen, The Netherlands; katherine.li@wur.nl (K.J.L.); elske.brouwer-brolsma@wur.nl (E.M.B.-B.); edith.feskens@wur.nl (E.J.M.F.); 2 Agroscope, Federal Office for Agriculture (FOAG), Federal Department of Economic Affairs, Education and Research (EAER), Schwarzenburgstrasse 161, CH-3003 Bern, Switzerland; carola.blaser@agroscope.admin.ch (C.B.); rene.badertscher@agroscope.admin.ch (R.B.); reto.portmann@agroscope.admin.ch (R.P.); guy.vergeres@agroscope.admin.ch (G.V.)

**Keywords:** dairy, milk, cheese, yoghurt, food intake biomarkers, multi-markers, validation

## Abstract

Studies examining associations between self-reported dairy intake and health are inconclusive, but biomarkers hold promise for elucidating such relationships by offering objective measures of dietary intake. Previous human intervention studies identified several biomarkers for dairy foods in blood and urine using non-targeted metabolomics. We evaluated the robustness of these biomarkers in a free-living cohort in the Netherlands using both single- and multi-marker approaches. Plasma and urine from 246 participants (54 ± 13 years) who completed a food frequency questionnaire were analyzed using liquid and gas chromatography-mass spectrometry. The targeted metabolite panel included 37 previously-identified candidate biomarkers of milk, cheese, and/or yoghurt consumption. Associations between biomarkers and energy-adjusted dairy food intakes were assessed by a ‘single-marker’ generalized linear model, and stepwise regression was used to select the best ‘multi-marker’ panel. Multi-marker models that also accounted for common covariates better captured the subtle differences for milk (urinary galactose, galactitol; sex, body mass index, age) and cheese (plasma pentadecanoic acid, isoleucine, glutamic acid) over single-marker models. No significant associations were observed for yogurt. Further examination of other facets of validity of these biomarkers may improve estimates of dairy food intake in conjunction with self-reported methods, and help reach a clearer consensus on their health impacts.

## 1. Introduction

Dairy products are widely acknowledged as an essential component of a healthy, diverse diet. Over six billion people consume milk and dairy products globally [1], and rely on these foods as a source of critical nutrients for growth, development, and disease prevention. However, studies linking dairy and dairy fat intake with cardiovascular disease and cardiometabolic conditions have yielded inconsistent findings, and there is still no consensus among different systematic reviews and meta-analyses on this matter [2,3,4]. Furthermore, several recent studies have indicated that fermented dairy products may be responsible for the cardioprotective effects of dairy foods (e.g., [5,6]). Fermentation of milk releases bioactive compounds, including some with anti-hypertensive and immunomodulatory properties, which can convey additional nutritive value [7]. Certain fermented dairy products, such as yoghurt, also contain live bacterial cultures that can modify the composition of the gut microbiota, thereby influencing the risk of developing obesity, type II diabetes, and general cardiovascular diseases [6,8,9,10].

For epidemiologists, a challenging but necessary task lies in capturing the ‘true’ intake of dairy products, such that its relationship with disease risk can be accurately portrayed. A major limitation of current dietary assessment tools [i.e., food frequency questionnaires (FFQ), 24-h recalls] is their reliance on subjective reporting by participants while food intake biomarkers (FIBs) offer an objective alternative, which can be used in conjunction with self-report tools to improve the dietary intake assessment of dairy food intake [11]. The odd-chain fatty acids pentadecanoic acid (C15:0) and heptadecanoic acid (C17:0) have been used as markers for total dairy intake (in particular, dairy fat), and have been effectively used for adjusting intakes when examining role of dairy consumption on cardiometabolic diseases [12]. However, these FIBs may not be as useful in capturing low-fat dairy products or distinguishing between specific dairy foods, and have also been criticized for being non-specific when assessing dairy intake in populations with high fish consumption [13]. In addition, given the limitations of using single biomarkers to assess dietary intake of a food (i.e., non-specific and high inter-individual variation), a multi-marker approach implying a combination of FIBs may improve the precision of the assessment [14]. Recently, multi-marker models have been developed for various foods including wine [15] and cocoa [16]. The sum of C15:0, C17:0, and/or trans-palmitoleic acid (t16:1n-7) have been previously used as biomarkers of dairy fat [17], but combined biomarkers reflecting the intake of specific dairy foods have not been exploited.

Eight criteria have been proposed for the validation of FIBs, one of which includes an evaluation of their robustness in both controlled intervention settings as well as free-living populations with complex, uncontrolled diets [18]. A number of previous acute and short-term, controlled human intervention studies have already been conducted in our laboratory and resulted in the identification of several FIBs for milk, cheese, and yoghurt, in serum and urine using untargeted metabolomics [19,20,21,22]. These FIBs were identified using a combination of LC-MS, GC-MS, and NMR, since each platform offers unique advantages for the detection of specific compounds based on factors such as compound size, polarity, abundance, and ionization, and their combined use permits complementary coverage of the metabolome [23]. In these studies, we also observed high inter-individual variability in the response of several candidate FIBs based on genetic variation. Specifically, postprandial responses for the lactose metabolites galactose, galactitol and galactonate in the serum and urine of healthy men following acidified milk intake were concordant with genetic lactase persistence [24]. In another study, we found that the oligosaccharides Lewis A trisaccharide and Blood Group H disaccharide reflected milk intake, and hypothesized that this was dependent on the expression of galactoside 2-alpha-L-fucosyltransferase 2 (FUT2) or galactoside 2-alpha-L-fucosyltransferase 3 (FUT3) enzymes, which act in competition to influence the production of these metabolites [20].

In the current paper, we aimed to evaluate the robustness of previously-identified candidate FIBs for milk, cheese, and yoghurt in a free-living population in the Netherlands, using both single- and multi-marker approaches, with investigation of known covariates and genetic targets. For comparison, we also evaluated the performance of C15:0 and/or C17:0 for predicting total dairy intake (as well as dairy intakes grouped by fermentation status and high/low fat content) in our population.

## 2. Results

### 2.1. Characteristics of the Validation Sub-Cohort

The characteristics of the validation sub-cohort is provided in Table 1. The majority of the participants were men (67%). The mean age of the participants was 54 ± 13 years, with men (56 years) being significantly older than women (51 years). A majority of men (63%) and almost half (46%) of women had a body mass index (BMI) corresponding to overweight or obese (≥25 kg/m^2^), which were also significantly different between sexes. A higher number and proportion of women (*n* = 12, 15%) than men followed a diet within the month preceding the study. The vast majority (95%) of participants were categorized as lactase persistent. Further, based on FUT2/FUT3 enzyme functional status, a majority of participants (with similar proportions in men and women) were classified as ‘secretors’ (79%), while a smaller percentage were ‘non-secretors’ (17%) and Lewis negative (4%).

### 2.2. Intake Levels of Different Dairy Products

Quintiles of median energy-adjusted intakes for different dairy groups and individual dairy foods are presented in Table 2. All participants consumed at least one type of dairy product, but some participants did not consume low-fat non-fermented dairy (*n* = 65), high-fat fermented dairy (*n* = 41), milk (*n* = 37), yoghurt (*n* = 34), high-fat non-fermented dairy (*n* = 9), cheese (*n* = 4), high-fat dairy (*n* = 3), total non-fermented dairy (*n* = 2), low-fat fermented dairy (*n* = 2), low-fat dairy (*n* = 1), and/or any fermented dairy (*n* = 1). Median intakes in the highest quintile of consumption (Q5) ranged from 60 g/d for high-fat non-fermented dairy to 527 g/d for total dairy. For individual dairy foods, median intakes were highest for milk (303 g/d), followed by yoghurt (193 g/d) and cheese (67 g/d). Sex-specific intake quintiles for the different dairy groups and individual dairy foods are presented in Tables S1 and S2 for men and women, respectively. Overall, men tended to have higher median intakes of high-fat dairy, total fermented dairy, high-fat fermented dairy, high-fat non-fermented dairy, low-fat fermented dairy, cheese, and yoghurt compared to women (in the majority of quintiles). However, women had higher median intakes of low-fat dairy, total non-fermented dairy, low-fat non-fermented dairy, and milk in all quintiles, as well as total dairy (in all except lowest quintile) compared to men.

### 2.3. Assessment of Biomarkers for Milk Intake

**Figure 1:**
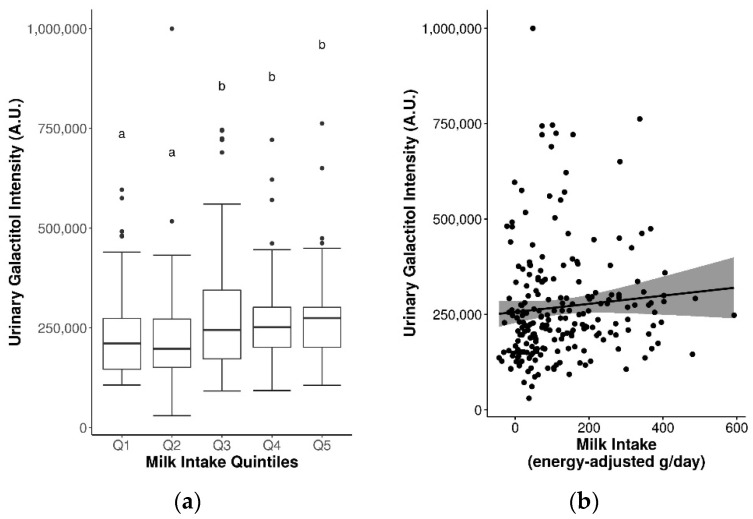
Significantly increased urinary galactitol levels by (**a**) quintiles of milk intake (significance between quintiles denoted by different letters, *p* ≤ 0.05), and (**b**) continuous milk intake.

Twenty-one candidate FIBs in Table S3 that were previously found to be discriminant for milk intake were assessed, of which fifteen were detected in plasma and/or urine. When analyzed by quintiles of milk intake, a statistically significant increase in urinary galactitol was observed (Q3–5 vs. Q1–2, *p* ≤ 0.05) (Figure 1a). Additional significant findings with an increasing trend were observed between FIBs and sex-specific quintiles of milk intake, including plasma phenylalanine and Lewis A trisaccharide in women, and urinary lactose and galactitol in men (Figure S1).

Spearman’s correlations were weak and non-significant for the majority of milk FIBs, with the exception of urinary lactose (r_s_ = 0.16, *p* ≤ 0.05) and galactitol (r_s_ = 0.2, *p* ≤ 0.05) (Table 3, Figure 1b). Some sex-specific correlations were also observed between plasma phenylalanine, tyrosine, tryptophan, indole-3-acetic acid, and Lewis A trisaccharide with milk intake in women, and urinary lactose and galactitol with milk intake in men (Table 3). These associations were paralleled in several single-marker generalized linear regression (GLM) models, with significant results observed for urinary lactose, galactose, and galactitol in covariate-adjusted models (coefficients = 0.07–0.20, r_ap_ = 0.17–0.2, R^2^ = 0.03–0.04, MAE ~ 93 g/d) (Table 3). Unadjusted and adjusted multi-marker models for milk derived from stepwise regression are presented in Tables S4 and S5. Adjusted multi-marker models consisting of urinary galactose + galactitol + sex + BMI + age (analyzed by GC-MS) [r_ap_ = 0.20, R^2^ = 0.04, mean absolute error (MAE) = 92 g/d], and plasma tryptophan + indole-3-propionic acid + sex [analyzed by liquid chromatography-mass spectrometry (LC-MS)] (r_ap_ = 0.25, R^2^ = 0.06, MAE = 102.8 g/d) had slightly improved performance accuracy for predicting milk intake compared to the single-marker models.

The role of lactase persistence status on the relative abundance of lactose and its metabolites in plasma and urine was further explored (Figure S2). As expected, plasma levels of lactose were low across all samples, due to the analysis of fasting samples, while urinary lactose was significantly higher in lactase non-persistent (LNP) individuals. While a higher relative abundance of all lactose metabolites was generally observed in lactase persistent (LP) individuals compared to LNP (with the possible exception of galactose in urine), the differences were not significant. Due to the low numbers of LNP individuals in our sub-cohort, further analyses of levels of lactose metabolites by quintiles of milk intake stratified by LP/LNP status was not possible.

Similarly, the role of FUT2 and FUT3 enzyme status on levels of Lewis A trisaccharide in plasma and Blood Group H disaccharide in plasma and urine was also explored (Figure S3). No significant differences were observed in plasma Lewis A trisaccharide between secretors and non-secretors. For Blood Group H disaccharide, while no significant between-group differences were detected in plasma (borderline *p* = 0.057), significantly higher levels were observed in urine for secretors compared to non-secretors (*p* = 2.5 × 10^−9^).

### 2.4. Assessment of Biomarkers for Cheese Intake

Sixteen previously-identified candidate FIBs for cheese intake are presented in Table S3, of which 14 were detected in plasma and/or urine samples of the current cohort (Table 4). For the total population, no significant differences in candidate FIB concentrations for cheese were observed across quintiles of cheese intake. However, when stratified into sex-specific intake quintiles, levels of urinary indole-3-lactic acid were significantly increased between quintiles of cheese intake in men, while plasma phenylalanyl-proline was increased between quintiles of cheese intake in women (Figure S4). For plasma proline in men, although also significant across quintiles, a decreasing trend was observed (Figure S4).

No significant positive Spearman’s correlations were observed between FIBs and cheese intake on a continuous scale for any of the FIBs, but when stratified by sex, a significant correlation was revealed for urinary indole-3-lactic acid in men (Table 4). No significant single-marker models (adjusted or unadjusted) were generated for any of the FIBs for cheese intake. From the multi-marker models, a combination of plasma C15:0 + isoleucine + glutamic acid [analysed by gas chromatography-mass spectrometry (GC-MS)] yielded a significant model for predicting cheese intake, albeit with somewhat poor performance (r_ap_ = 0.16, R^2^ = 0.03, MAE = 17 g/d) (Table S4). Inclusion of covariates in an adjusted multi-marker model did not further reveal combinations of biomarkers or biomarker and covariates that better predicts cheese intake (plasma C15:0 + isoleucine + glutamic acid was still the best model) (Table S5).

### 2.5. Assessment of Biomarkers for Yoghurt Intake

Out of the ten candidate FIBs that were previously-identified for yoghurt intake in plasma (Table S3), eight were detected in plasma in the current study (Table 5). No significant differences were found between plasma levels of these FIBs by increasing quintiles of yoghurt intake in the total population. However, when stratified into sex-specific intake quintiles, a significant difference was found for plasma tyrosine in women (Q2–5 vs. Q1, *p* ≤ 0.05), although it should be noted that Q1 comprised primarily non-consumers (Figure S5).

Spearman’s correlations were weak and non-significant for all FIBs. Similarly, there were no significant single-marker models for yogurt (unadjusted or adjusted) (Table 5). From the multi-marker models, a significant adjusted model consisting of threonine + tyrosine + sex was generated for yoghurt intake (Table S5). However, the model performance was very poor (r_ap_ = 0.03, R^2^ = 0.0008, MAE = 68 g/d).

### 2.6. Assessment of Pentadecanoic Acid (C15:0) and Heptadecanoic Acid (C17:0) as Biomarkers for General Dairy Intake

**Figure 2:**
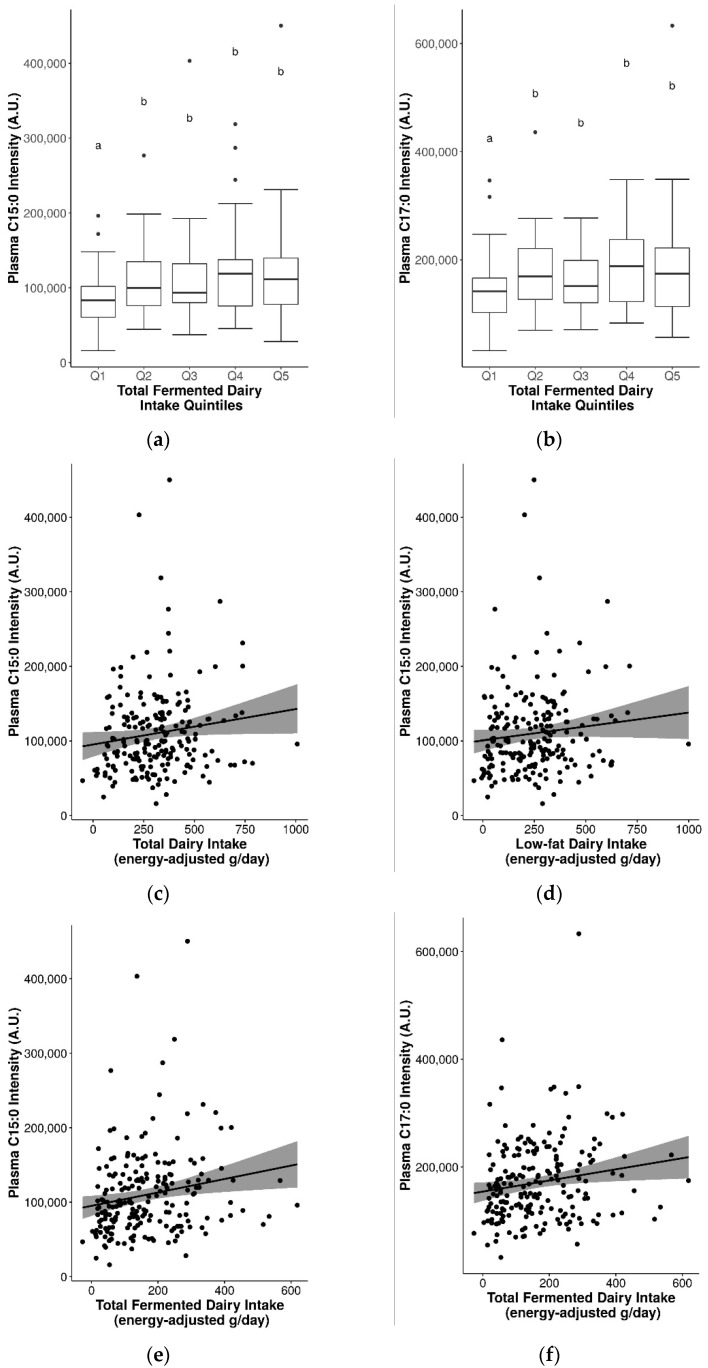

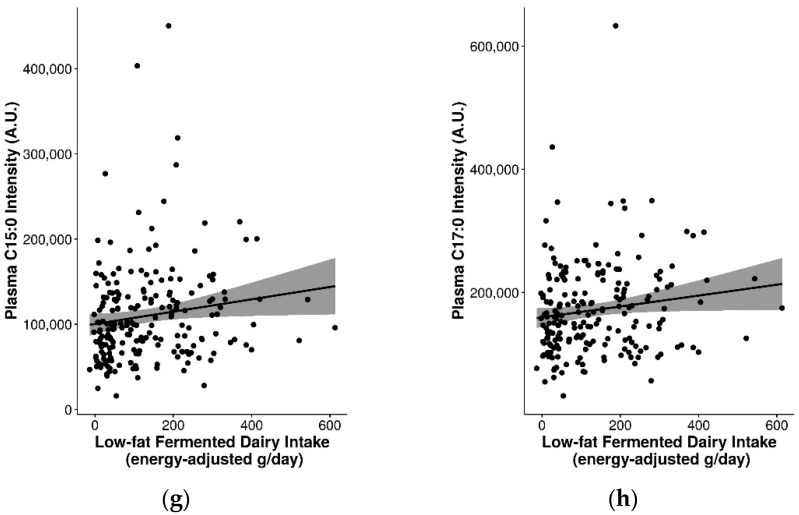
Significantly increased plasma pentadecanoic acid (C15:0) and/or heptadecanoic acid (C17:0) with increasing dairy intake. (**a**) C15:0 by quintiles of total fermented dairy intake, (**b**) C17:0 by quintiles of total fermented dairy intake (significance between quintiles denoted by different letters, *p* ≤ 0.05), (**c**) C15:0 by continuous total dairy intake, (**d**) C15:0 by continuous low-fat dairy intake, (**e**) C15:0 by continuous total fermented dairy intake, (**f**) C17:0 by continuous total fermented dairy intake, (**g**) C15:0 by continuous low-fat fermented dairy intake, (**h**) C17:0 by continuous low-fat fermented dairy intake.

Differences in the relative abundance of C15:0 and C17:0 in fasting plasma were assessed by quintiles of intake for various dairy groups. Significantly higher C15:0 and C17:0 were observed with higher quintiles of total fermented dairy intake (Q2-Q5 vs. Q1, *p* ≤ 0.05) (Figure 2a,b), but not for other dairy groups. For C17:0 and total non-fermented dairy intake, the effect was not clear (Q1, Q3, Q5 vs. Q2, Q4 vs. Q3, *p* ≤ 0.05) (Figure S6). In addition, no significant differences in levels of these fatty acids were observed between intake quintiles for other dairy groups (including total dairy), even when stratified into sex-specific intake quintiles.

The seemingly stronger links between these biomarkers and fermented dairy groups was further observed in analyses with continuous intakes. Although correlations between C15:0 or C17:0 with dairy groups were generally weak, they were positive and significant for total dairy (C15:0 r_s_ = 0.17), low-fat dairy (C15:0 r_s_ = 0.16), total fermented dairy (C15:0 r_s_ = 0.24; C17:0 r_s_ = 0.19), and low-fat fermented dairy (C15:0 r_s_ = 0.19; C17:0 r_s_ = 0.16) (*p* ≤ 0.05) (Table 6 and Figure 2c–h). When stratified by sex, significant positive correlations were observed in men for C15:0 and C17:0 with low-fat dairy, total fermented dairy, and between C15:0 and low-fat fermented dairy. Similarly, in the single-marker regression models, positive and significant models were generated for C15:0 and total dairy, total fermented dairy, and low-fat fermented dairy intake, and similarly for C17:0 and total and low-fat fermented dairy intake (Table 6). Adjustment of the models by sex, BMI, and age also revealed a significant model for C15:0 and low-fat dairy. For C15:0 and total dairy intake, adjustment measurably improved the model performance (r_s_ = 0.3, R^2^ = 0.1, MAE = 125 g/d).

The best multi-marker models for C15:0 and C17:0 derived from stepwise regression for dairy foods and dairy groups are presented in Table S6. In the unadjusted models, C15:0 alone was revealed to be the best predictor of intakes for all dairy groups, and was significant for total dairy, total fermented dairy, and low-fat fermented dairy. In the adjusted models, the best (most parsimonious) models were generated from a combination of biomarker/covariates, and consisted of C15:0 + sex + BMI for total dairy, low-fat dairy, and total non-fermented dairy, C15:0 + sex for total fermented dairy, and C15:0 + age for low-fat fermented dairy. For low-fat non-fermented dairy, the best model did not include a biomarker but was instead driven by two covariates (sex + BMI). C15:0 was positively associated with dairy intakes in all models, while covariates were negatively associated. In all cases, the adjusted model selected from stepwise regression had the best model outcomes (in terms of model significance and prediction performance); however, this did not involve a true multi-marker combination consisting of C15:0 + C17:0. It is noteworthy that a high degree of multicollinearity between C15:0 and C17:0 was observed [variance inflation factor (VIF) > 5, r = 0.93)].

### 2.7. Suitability of Biomarkers for Discriminating between Fermented and Non-Fermented Dairy Intake

**Figure 3:**
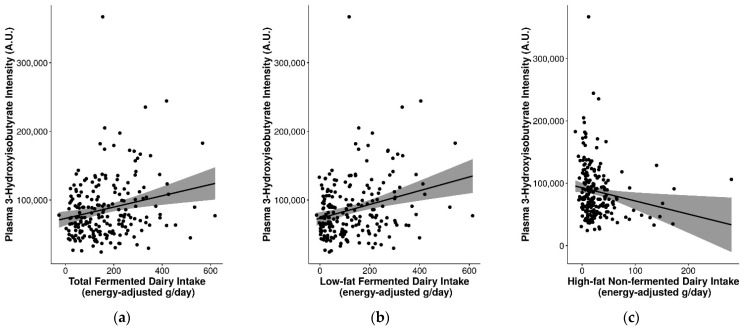

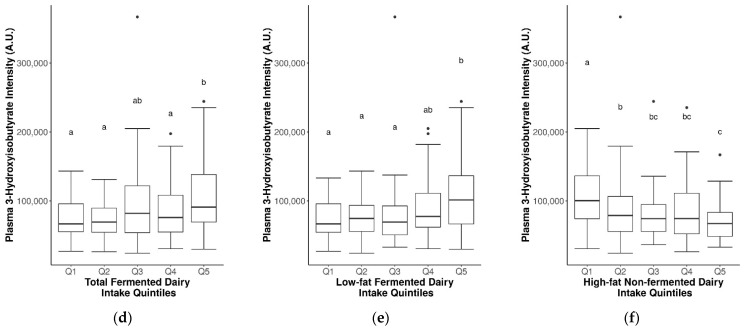
Significant differences in plasma 3-hydroxyisobutyrate levels between fermented and non-fermented dairy groups. Analyzed by continuous intake or intake quintiles of (**a**,**d**) total fermented dairy, (**b**,**e**) low-fat fermented dairy intake, and (**c**,**f**) high-fat non-fermented dairy intake. Significance between quintiles denoted by different letters (*p* ≤ 0.05).

Several of the FIBs had significant positive correlations with fermented dairy intake and/or negative correlations with non-fermented dairy intake (Table S7). In particular, consistent correlations were observed for plasma 3-hydroxyisobutyrate (r_s_ = 0.2 for total fermented dairy, r_s_ = 0.23 for low-fat fermented dairy intake, r_s_ = −0.24 for high-fat non-fermented dairy intake; *p* ≤ 0.05) and were reflected in the different levels of 3-hydroxyisobutyrate for the different quintiles of intake for these dairy groups (Figure 3). Other relevant markers included plasma C15:0, C17:0, galactonate, lactose, valine, galactitol, 3-phenyllactic acid, glutamic acid, isoleucine, leucine, methionine, and proline (detected with GC-MS) (Figure 3 and Figure S7). These FIBs were included in an exploratory multi-marker model to gauge whether their inclusion can help to better predict fermented and non-fermented dairy intake.

As seen in Table S8, when compared to Table S6, multi-marker models with these FIBs improved the model performance of the majority of dairy groups compared to models with C15:0 and/or C17:0. The best multi-marker models selected for total fermented dairy (C15:0 + galactonate + glutamic acid + lactose + methionine + 3-hydroxyisobutyrate; unadjusted, r_ap_ = 0.22, R^2^ = 0.05, MAE = 101 g/d), high-fat fermented dairy (C15:0 + 3-hydroxyisobutyrate + BMI; adjusted, r_ap_ = 0.4, R^2^ = 0.16, MAE = 35 g/d), and low-fat fermented dairy (C15:0 + lactose + 3-hydroxyisobutyrate + galactonate + glutamic acid + methionine; unadjusted, r_ap_ = 0.25, R^2^ = 0.06, MAE = 93 g/d) all included significant positive associations with C15:0. Furthermore, 3-hydroxyisobutyrate was selected in the best model for all three fermented dairy groups: positively associated for total (non-significant) and low-fat fermented dairy (significant), and negatively associated with high-fat fermented dairy (significant). The best performing models for non-fermented dairy groups included valine + 3-hydroxyisobutyrate + 3-phenyllactic acid + BMI + sex for total non-fermented dairy (adjusted, r_ap_ = 0.02, R^2^ = 0.00, MAE = 93 g/d), C17:0 + isoleucine + leucine for high-fat non-fermented dairy (unadjusted and adjusted, r_ap_ = 0.4, R^2^ = 0.16, MAE = 27 g/d), and valine + 3-hydroxyisobutyrate + 3-phenyllactic acid + sex + BMI for low-fat non-fermented dairy (adjusted, r_ap_ = 0.11, R^2^ = 0.01, MAE = 96 g/d). For total and low-fat non-fermented dairy, 3-hydroxyisobutyrate was significantly and negatively associated with intake in adjusted models. Furthermore, inclusion of these candidate FIBs also improved the prediction of high-fat and low-fat dairy groups (r_ap_ = 0.27–0.5, R^2^ = 0.07–0.25) (Table S8).

## 3. Discussion

In the current study, we aimed to evaluate the robustness of the previously-identified candidate FIBs for milk, cheese, and yoghurt. Most of the selected biomarkers have already shown some of the essential qualities of a FIB including plausibility and time-response in a controlled intervention setting [18,19,20,21,22], but observational data in free-living populations is limited. The single-marker models examined in this observational study did not perform well in predicting the intake of dairy foods in our free-living population, which may be related to the fact that these FIBs are non-specific and can be influenced by consumption of other foods in the diet. However, we observed modest associations for multi-marker models that also account for known covariates, suggesting that they may help better capture the subtle differences between specific dairy foods. Moreover, our analyses illustrate several challenges and considerations critical to further validation of these FIBs.

### 3.1. Biomarkers for General Dairy Intake, Dairy Food Intake, and Their Specificity

By far, the most common dietary biomarkers described in studies of dairy intake are C15:0 and C17:0. Despite their widespread use, several limitations have been acknowledged, including their non-specificity for dairy in populations with high fish intake due to their endogenous presence in fish [13]. Furthermore, although C15:0 has been suggested to be an effective concentration biomarker of dairy intake in controlled animal studies, only moderate correlations have been reported in human observational studies [25]. Due to these limitations, as well as the inability of these biomarkers to discriminate between specific dairy foods, the identification of further FIBs for dairy products is a valuable endeavour. A previous systematic review on biomarkers of dairy products identified several plausible FIBs of total dairy intake, including serum C15:0, C17:0, C17:1, myristoyl-sphingomyelin SM (d18:1/14:0), and galactonate, as well as urinary isovalerylglutamic acid, isovalerylglycine, tiglylglycine, and isobutyrylglycine for cheese intake [26]. No specific biomarkers were identified for yoghurt consumption.

In the present study, we evaluated the association of C15:0 and C17:0 in fasting plasma with dairy intake, the results of which helped contextualize the associations and validation performances of the other FIBs. Although associations were generally low (r_s_ = 0.16–0.24), they were comparable to observational studies with similar study designs (r = 0.1–0.36) [27,28,29]. Other FIBs we aimed to evaluate for milk, cheese, and yoghurt [19,20,21,22] were mainly non-significant, or if significant, yielded weak positive associations. This may be partly due to the presence of the FIBs or their parent compounds in different foods. For instance, while lactose is the predominant carbohydrate in milk, its presence in commonly-consumed processed foods containing milk ingredients may obscure the specificity of lactose and its metabolites for assessing milk intake [30]. Additionally, the majority of FIBs for cheese and yoghurt (peptides, amino acids, and their intermediates) can also be influenced by the consumption of a large variety of protein-rich foods in the diet. The single-marker validation of these non-specific FIBs in a free-living population presents a tremendous challenge, but their inclusion in a multi-marker panel appears to be more promising.

### 3.2. Single- versus Multi-Marker Models for Evaluating the Robustness of FIBs

Since milk is a complex mixture of macronutrients, micronutrients, minerals, and bioactive compounds, it is intuitive to seek out multiple biomarkers to capture and discriminate the intake of milk and dairy products. By using regression models, we could assess and compare the ability of single- vs. multi-marker approaches in predicting intake of specific dairy foods and dairy groups. Selected physiological covariates that can affect and/or be affected by the choice of dairy food consumed as well as absolute intake levels and patterns of consumption (sex, age, and BMI) [31] were also included in the biomarker models.

In our models, C15:0 performed better than C17:0 for predicting general dairy intake in both single- and multi-marker models, confirming what has been previously observed in the literature. From the single-marker models, urinary lactose, galactose, and galactitol were the most effective FIBs in predicting milk intake (better than C15:0 or C17:0), while two adjusted multi-marker models (galactose + galactitol + age + sex + BMI; indole-3-propionic acid + tryptophan + sex) offered slightly improved prediction performance. Galactose, galactitol, and tryptophan were positively associated with milk intake in these models, but indole-3-propionic acid (a deaminated metabolite of tryptophan) was negatively associated with milk intake. While milk consumption previously generated a significant postprandial increase in indole-3-propionic acid [21], it was not detected in milk, suggesting that indole-3-propionic acid may have been synthesized from tryptophan in milk by the gut microbiota [32].

A significant multi-marker model consisting of plasma C15:0, isoleucine and glutamic acid captured cheese intake, whereas no significant single-marker models were generated. As cheese products tend to be higher in dairy fat (compared to milk and yoghurt), it is not surprising that C15:0 was selected in the multi-marker model for cheese. Similarly, glutamic acid has been previously reported as the primary compound responsible for the ‘umami’ taste quality of cheese products [33]. Conversely, isoleucine is ubiquitous in the diet, and cheese consumption may not have been sufficient to impact isoleucine levels significantly. This is reflected in the non-significant but negative correlation between plasma isoleucine and cheese intake, and the negative association in the regression model. For yoghurt intake, no significant single-marker models were generated. A significant adjusted multi-marker model comprising threonine and tyrosine was generated for yoghurt intake, however, the model performance was low, perhaps due to the non-specific nature of the panel of FIBs for yoghurt (primarily amino acids).

### 3.3. Evaluation of Other Facets of Validity

In the case of non-specific biomarkers, a major factor affecting intake-biomarker associations is the quantity of food consumed. The Netherlands has one of the highest per capita dairy consumption, which makes our population highly suitable for evaluating dairy biomarkers. However, within dairy foods, consumption of cheese was comparatively lower than consumption of milk in our population (median ~ 27 vs. 87 g/d), with a narrower range of intakes (8 to 67 g/d vs. 4 to 303 g/d in Q1 to Q5). This can affect the ability of FIBs to discriminate between individuals with high or low intakes, and blurs the dose-response relationship. Although true dose-response could not be evaluated in our study, we observed significant increases in multiple FIBs across quintiles of dairy food intake, in particular, urinary galactitol for milk intake. Several FIBs also showed apparent sex-related responses in the stratified quintile analysis and correlations. These findings could be affected by the differences in numbers of participants between sexes, which may have afforded higher statistical power in men (*n* ~ 33 per quintile) rather than women (*n* ~ 16 per quintile).

We also acknowledge that the composition of (bovine) milk can be affected by animal grazing conditions, which could lead to seasonal variations in levels of biomarkers in the blood or urine. A study conducted in the Netherlands reported that the most pronounced differences in milk composition were in fatty acid concentrations (decrease in saturated fatty acids and increase in trans fatty acids during the grazing season, ~April–September), while lactose and protein composition remained relatively stable [34]. Similar effects on other metabolites/biomarkers are unknown.

Other validation criteria of reliability, stability, analytical performance, and reproducibility could not be sufficiently addressed here, but a few related considerations are worth noting. For biomarker discovery, the combined use of multiple metabolomics analytical platforms (e.g., LC-MS, GC-MS, and NMR) permits complementary coverage of the metabolome and is particularly valuable for identifying unique sets of FIBs based on individual platform strengths [23]. For validation, targeted platforms are often favoured, to quantify a limited panel of compounds but often with improved methodology for a specific compound class. In the case of dairy fatty acids (including C15:0 and C17:0), the most widely used methodology for their separation and analysis is a targeted, quantitative method using chromatography-flame ionization detector (GC-FID) [35]. Thus, further method development and quantitative analyses of these fatty acids as well as other FIBs for milk, cheese, and yogurt may improve their performance for estimating dairy (food) intake in a multi-marker model, along with their reliability and analytical performance.

Another important consideration consequential for successful FIB validation is that the choice of biosample may reflect a different time-course associated with intake. Long-term fat intake is best measured using adipose tissue (1–2 years), whereas short-term intake is best assessed using serum phospholipids or cholesteryl esters (past several days) and triglyceride fractions (past several hours) [36,37,38]. In the present study, we used fasting plasma and 24-h urine samples that were banked and readily available for analysis. FIBs with short half-lives in plasma were unsurprisingly not significant. For example, in the metabolomics study of yoghurt intake, significant increases in several compounds were observed in postprandial plasma, but almost all were not significant in fasting serum after daily yoghurt intake for two weeks [21]. For metabolites measured in both plasma and urine (e.g., lactose, Blood Group H disaccharide), higher relative abundance was observed in urine samples that were collected over 24 h whereas levels were almost undetectable in plasma samples collected under fasting conditions. While these FIBs may not be suitable as markers of habitual intake of dairy foods, they may still be valid as markers of short-term or recent intake. Therefore, further exploration of the FIBs outlined in this study in several other independent observational and intervention studies using samples with different time courses would help assess their robustness as short-term biomarkers.

### 3.4. Influence of Fat Content and Fermentation on Dairy Biomarkers

One objective in the current study was to explore the potential influence of food-related factors on the efficacy of dairy biomarkers, in particular fat content and fermentation status. Our analyses revealed significant positive associations between plasma C15:0 with total and low-fat dairy intake, and between C15:0 and C17:0 with total and low-fat fermented dairy intake, but similar anticipated results were not observed for high-fat dairy groups. One explanation could be the generally lower consumption of high-fat dairy products (Q1: 10 g/d to Q5: 135 g/d) compared to low-fat dairy products (Q1: 43 g/d to Q5: 480 g/d) in our population. While lower intakes inherently translates to lower concentrations of candidate FIBs in biofluids, the group of high-fat dairy foods also tended to include more sporadically consumed products (e.g., cream with hot meal, whipped cream, milk-based ice cream). A combination of these factors can introduce variability and error. Further, there is a small possibility that these fatty acids are enriched in fermented dairy products, as fermentation of milk has been shown to impact the fatty acid profiles of cheese and yoghurt products [39].

From our exploratory analyses assessing the suitability of FIBs for discriminating between fermented and non-fermented dairy intake, a significant positive association was found between 3-hydroxyisobutyrate and total fermented dairy and low-fat fermented dairy intake, and simultaneously inversely associated with high-fat non-fermented dairy as well as high-fat non-fermented intake, suggesting an overall positive association between this FIB and fermented dairy. This association was also partly reflected in the multi-marker models for fermented and non-fermented dairy groups, although not fully confirmed as the direction of association of 3-hydroxyisobutyrate with the various dairy groups presented a complex pattern. Further, 3-Hydroxyisobutyrate is synthesized in the rumen of dairy cattle via the action of butyrate-producing bacteria, and also in ketogenesis as a catabolic product of valine [40]. Further studies are needed to strengthen the biological plausibility of this finding, and drive efforts to identify FIBs related to fermentation that will help elucidate the underlying mechanisms for fermented dairy consumption and cardiometabolic health.

### 3.5. Influence of Genetic Variants on Biomarkers of Milk Intake

Genetic polymorphisms in key enzymes leads to inter-individual variability in the metabolism of a compound, thereby impacting its efficacy and limiting its capacity as a quantitative biomarker. For dairy foods, a dominant mutation in the lactase enzyme (especially LCT-13910 C > T) is critical for lactose metabolism in adulthood [41]. The global prevalence of lactase persistence is highly geographically dependent (e.g., <1% in Asia, >90% in Northern Europe) [41], and in our study population comprising primarily Caucasian Dutch adults, the level of lactase persistence was very high (~95%). This resulted in an uneven distribution between LP and LNP individuals (104 vs. 6), and the effects of lactase persistence on the efficacy of lactose metabolites as FIBs of milk intake could not be evaluated in this study with sufficient statistical power. However, in studies involving larger populations or comprising different ethnic populations, the presence of these genetic variants may be magnified, which could affect the predictions/accuracy of these FIBs and warrants careful consideration.

We also previously observed high inter-individual variation in two Lewis system-related oligosaccharides, Lewis A trisaccharide and Blood Group H disaccharide, identified as potential FIBs of (bovine) milk intake [20]. In humans, the production of these fucosylated oligosaccharides is determined by expression of the *FUT2* and *FUT3* genes [20]. The majority of individuals with functional FUT2 are deemed ‘secretors’, while those who inherit a homozygous loss-of-function mutation are deemed ‘non-secretors’ [42]. The non-secretor phenotype (~20% of Caucasians) has been associated with higher susceptibility to various gastrointestinal diseases and infections [42,43,44,45]. Non-secretors with a functional FUT3 enzyme can still express Lewis A antigens, but in rare cases, mutations of both *FUT3* alleles results in the Lewis negative phenotype (6%) [20,42]. In the present study, a comparable prevalence of secretors, non-secretors, and Lewis negative was observed (79%, 17%, and 4%, respectively). As expected, significantly higher urinary Blood Group H disaccharide was found in FUT2 secretors; an increase was also observed in plasma but was only of borderline significance, presumably due to low overall concentrations. These metabolites were not found to be discriminant for milk intake in our study, which could be attributed to their largely endogenous origin; nonetheless, attention in larger studies will help clarify their classification and impact as FIBs for milk.

### 3.6. Study Limitations

There are several limitations worth noting. Firstly, based on the data available, we relied on a window of ±14 days between biosample collection and the completion of an FFQ, which assesses habitual intake of the previous month. This assumes that dietary consumption patterns the day prior to biosample collection were comparable to the reported intakes, but otherwise, would be a source of measurement error. Secondly, since the FIBs were identified as part of a larger non-targeted study, we relied on metabolite relative abundances instead of absolute quantitative data for the validation, which limits the ability for data integration between analytical platforms. Thirdly, like other studies in metabolomics epidemiology, we used pre-existing biosamples from large observational studies that were not originally designed for the purpose of metabolomics analyses, and sample incubation time could influence levels of certain metabolites. Fourthly, the dairy products administered in the intervention studies where the FIBs were derived from may have a different compositional profile than those consumed in the free-living cohort. In the particular case of cheese, all FIBs were identified following consumption of Swiss Gruyère cheese, whereas consumption of Dutch cheeses (Edam, Gouda) are predominant in the current study population. Finally, we relied on generalized linear and stepwise regression models for comparing single- and multi-marker validation results, and in particular, for determining the predictive ability of the FIBs. Aside from limitations inherent to regression models (e.g., multicollinearity), we acknowledge that these FIBs may perform better in predicting ranked intakes or binary outcomes (i.e., extreme quintiles). Since we wanted to evaluate the robustness of these biomarkers using the full population, we used a continuous approach, which also permitted comparison to previous studies conducted for C15:0/C17:0. Further use of quantitative data for the strongest biomarker models will further assess agreement between biomarker-based and subjective reporting methods.

## 4. Materials and Methods

### 4.1. Study Population

The Nutrition Questionnaires Plus (NQplus) study is a prospective cohort study that was conducted in Dutch adults (primarily Caucasian, 20 to 70 years), living in or around Wageningen (the Netherlands). NQplus was initiated as an ‘add-on’ study to the National Dietary Assessment Reference Database (NDARD) project, to gather extensive data on participant demographics, lifestyle, medical history, and cardiometabolic health outcomes. A complete description of NQplus and NDARD has been provided elsewhere [46,47]. Briefly, 2048 men and women were recruited and included in the study between June 2011 and February 2013. Baseline measurements included an assessment of habitual dietary intake by FFQ and/or 24-h recall. Background demographics, health, anthropometric, and lifestyle data, along with fasting blood samples [total collected: EDTA plasma (6 × 0.5 mL + 1 × 1.5 mL), citrate plasma (5 × 0.5 mL), serum (3 × 0.5 mL + 2 × 1 mL) and one buffy coat sample] and 24-h urine samples (mean ± SD weight: 2282 ± 814 g), were also collected. All biosamples were stored in the biobank at −80 °C for future analysis. All measurements were performed according to a standardized protocol by trained research personnel. The study was approved by the ethical committee of Wageningen University and Research (protocol number NL34775.081.10) and conducted in agreement with the Declaration of Helsinki. Written informed consent was obtained from all participants prior to the start of the study.

Metabolomics analyses were performed on a sub-cohort of NQplus participants (*n* = 531), including participants with ‘complete’ dietary data (completion of one FFQ and at least two 24-h recalls) as well as a biosample collected within 14 days of completing either a FFQ or a 24-h recall. For the present study, we report on *n* = 246 participants who had a biosample collected within ±14 days of completing a FFQ (228 plasma and 216 urine samples). This criterion ensured that biosample collection occurred within the FFQ reference period of one month, providing an assessment of typical dietary intake that is not as sensitive to fluctuations in daily intake as repeated 24-h recall assessments.

### 4.2. Food Frequency Questionnaire and Levels of Dairy Food Consumption

A full description of the FFQ used to assess habitual dietary intake has been described in the study design papers for NQplus and NDARD [46,47]. The FFQ was self-administered and completed online using the open-source survey tool LimeSurvey (LimeSurvey Project Team/Carsten Schmitz, Hamburg, Germany), with ten frequency categories ranging from ‘never’ to ‘6–7 days per week’. Portion sizes were estimated using commonly used household measures. Total food intake (in g/d) was determined by multiplying consumption frequency by portion size as defined in the Dutch food composition tables [48]. The majority of FFQ assessments were completed in the spring (*n* = 129) and summer (*n* = 94) months, with fewer assessments performed in the autumn (*n* = 9) or winter (*n* = 14) months. Although the intake levels of some dairy foods could be dependent on season, we did not observe a consistent trend for such differences. Out of 216 total food items in the FFQ, 39 were identified as dairy products, which were further classified into milk, cheese, yoghurt, cream, butter, buttermilk, quark, and ice cream subgroups (Table S9). This FFQ has been previously validated for energy, fat, and various nutrients and food groups [49,50,51], including milk, yoghurt, cheese, total fermented dairy, and total non-fermented dairy (against multiple 24-h recalls) [52], which were used in the current study for evaluation of the respective candidate FIBs.

In addition, to evaluate the performance of C15:0, C17:0, and various biomarkers on dairy groups, a total dairy group was calculated from the combined intakes of all dairy products, a total fermented dairy group was calculated from the combined intakes of all fermented dairy products in the FFQ, and a total non-fermented dairy group was calculated from the combined intakes of all non-fermented dairy products in the FFQ. Ingredient lists of common grocery store items were consulted (where necessary) to ensure that specific dairy foods were truly fermented, and composite dishes containing a fermented dairy ingredient (e.g., pizza with cheese) were excluded, as previously described [52]. Total dairy, fermented dairy, and non-fermented dairy groups were further stratified into high-fat groups, which included all full-fat dairy products, and low-fat groups, which included semi-skim and skim dairy products (Table S9). Fat content (g/100 g) for all dairy products was determined based on the values reported in the Dutch Food Composition Table [48] and classifications of products as skim, semi-skim, and full-fat were based on the guidelines set by the Dutch Dairy Commodities Act (see Table S9).

### 4.3. LC-MS Sample Preparation and Analysis

Plasma and urine samples were analyzed using liquid chromatography-mass spectrometry (LC-MS) and gas chromatography-mass spectrometry (GC-MS). All samples were thawed on ice and kept at 4 °C during analysis. Prior to LC-MS analysis, phospholipids were removed from plasma samples to limit ion suppression using the Phree filter (Phenomenex Inc., Torrance, CA, USA). Urine samples were normalized based on the specific gravity as determined by the refractive index (refractometer RE40, Mettler Toledo, Switzerland), as described in Pimentel et al. [20]. Briefly, urine samples were centrifuged at 1800× *g* for 10 min at 4 °C. The supernatant was then diluted using milliQ water to a specific gravity of 1.0008 to ensure that sample measurement occurred within the linear dynamic range of the machine. LC-MS metabolomics analysis was performed using the UltiMate 3000 RS UPLC system (Thermo Fisher Scientific, Waltham, MA) with a Waters Acquity UPLC HSS T3 column (length 150 mm, diameter 2.1 mm, particle size 1.8 µm), coupled with the maXis 4G + quadrupole time-of-flight mass spectrometer (Bruker Daltonik GmbH, Bremen, Germany). We ran a gradient from 5% to 95% of mobile Phase A within 15 min at 0.4 mL/min. Mobile Phase A consisted of Milli-Q water with 0.1% formic acid and mobile Phase B consisted of acetonitrile with 0.1% formic acid. The column was heated to 35 °C with a post column cooler set to 25 °C. The resulting system pressure was ~600 bar, dependent on the actual composition of the mobile phase at the specific time. The mass spectrometer electrospray interface was operated in positive ion mode and spectra were recorded from 75 to 1500 *m*/*z*. Collision-induced dissociation was performed using energies from 20 to 70 eV. A total of 5 uL of de-phosphoralized plasma or normalized urine from each sample were injected. All samples were injected once. Quality control (QC) pools were prepared from plasma or urine samples by mixing all samples of each sample type at equal volume. QC samples were injected at five sample intervals for signal drift correction. Blanks (consisting of ultrafiltered LC-MS-grade water) were also injected at the beginning and end of each batch for detection of contaminants. Progenesis QI (v.2.3.6198.24128, NonLinear Dynamics Ltd., Newcastle upon Tyne, UK) was used for retention time correction, peak-picking, deconvolution, adducts annotation, and normalization (default automatic sensitivity and without minimum peak width). The intensity and the detection limit of the candidate FIBs was also performed by Progenesis QI with the setting “default”. The software does not limit the detection at a certain intensity, but respects the noise level and presence of an isotopic pattern.

### 4.4. GC-MS Sample Preparation and Analysis

Plasma and urine samples were prepared for GC-MS analysis as previously described [19,22]. Urine samples were normalized prior to analysis using the refractive index methods described above for the LC-MS analysis. For each 100 µL plasma sample, 50 µL of an internal standard solution (labelled D-fructose, U-13C6, 99%, Cambridge Isotope Laboratories, Inc., Cambridge, UK, c ≈ 0.16 mg/mL in water) was added, followed by precipitation with 300 µL cold methanol, centrifugation, transfer of supernatant (370 µL), and drying using a vacuum centrifuge. For each 100 µL urine sample, 50 µL of an internal standard solution (labelled D-fructose) was added and dried using a vacuum centrifuge. The samples further underwent a two-step derivatization (methoximation with O-methylhydroxylamine hydrochloride followed by silylation with N-methyl-N-(trimethylsilyl)trifluoroacetamide (MSTFA)) and subjected to analysis on a GC-MS 7890B/MS5977A (Agilent Technologies, Santa Clara, CA, USA) with a CombiPAL autosampler (CTC-Analytics AG, Zwingen, Switzerland) and a DB-5 ms fused silica capillary column (60 m, 0.25 mm i.d., 0.25 μm film thickness, Agilent Technologies, Basel, Switzerland). The samples were injected using a multimode injector according to the following temperature program: initially 90 °C, heating rate 900 °C/min until 280 °C, hold for 5 min and cooled at rate of −30 °C/min, and kept at 250 °C. The oven program was as follows: initial temperature 70 °C for 2 min, increase up to 160 °C at a rate of 5 °C/min, increase to 300 °C at a rate of 10 °C/min, which was held for 36 min, equilibration time 1 min. MS detection mass ranged from 28.5 to 600 Da, MS source temperature was 230 °C, and MS Quad temperature was 150 °C. Electron ionization was performed with 70 eV. QC samples were prepared beforehand by mixing all plasma samples at equal volumes. Each batch was initiated by five injections of QC samples for equilibration and after every fifth plasma sample a fresh QC was injected. At start and end of each batch, a blank sample (milliQ water) was included. QC samples and blank samples underwent the same sample preparation as plasma samples.

Agilent data files acquired from GC-MS analysis were deconvoluted and converted into CEF files using Agilent Masshunter Profinder (Agilent Technologies, Santa Clara, CA, USA). Data files were further processed in Agilent Mass Profiler Professional (Agilent Technologies, Santa Clara, CA, USA) to perform, alignment and compound identification. In the resulting list containing the deconvoluted features, features with retention time before 10 min were removed (reagents region). All markers selected based on deconvoluted data were further evaluated using a targeted approach in order to optimize integration. Using RI, quantifier and qualifier ion retrieved from deconvoluted data, the suggested markers were analyzed in MassHunter Quantitative Analysis (Agilent Technologies, Santa Clara, CA, USA). The peak integration was checked in each sample individually. Responses from the quantifier ion of marker compounds were normalized with the response of the quantifier ion of internal standard labelled d-Fructose Peak 1 (ion 279).

### 4.5. Previously-Identified Candidate Biomarkers, Analytical Standards and Reagents

Candidate FIBs for milk, cheese, and yogurt were previously identified in serum and urine using non-targeted metabolomics, where the most discriminant FIBs were selected using Projections to Latent Structures Discriminant Analysis (PLS-DA) (details and figures reported elsewhere) [19,20,21,22]. A list of these FIBs is provided in Table S3. Where possible, we aimed to evaluate these previously-identified candidate FIBs for milk, cheese, and yoghurt in the biosample and using the same analytical platform by which they were originally identified. FIBs that were previously identified in serum were targeted in plasma. FIBs that were previously identified using NMR could not be assessed by the same platform in the present study; thus, we used GC-MS as a substitution platform for the identification of most of these FIBs. All solvents and reagents for metabolomics analysis were purchased from Sigma-Aldrich Chemie GmbH (Buchs, Switzerland).

For LC-MS, the Human Metabolome Database [53] and the National Institute of Standards and Technology database (NIST v14) were used to screen the identity of metabolites with a 10 ppm mass accuracy threshold. Compound identities were then confirmed with the injection of authentic standards with a RT window of 20%. A list of all standards suppliers is provided in Table S10. For GC-MS, an internal database was used for identification of targeted biomarkers. In the case that stereoisomeric forms of selected discriminating features were identified, the peak with higher response was further evaluated. Details of the identification features of compounds analyzed from LC-MS and GC-MS are presented in Tables S11 and S12, respectively.

### 4.6. Determination of Lactase, FUT2, and FUT3 Expression

Since the digestion of lactose and levels of lactose metabolites (galactose, galactonate, galactitol, galactono-1,5-lactone) are dependent on the presence of a functional lactase enzyme in adults [24], we evaluated the prevalence of the lactase persistent genotype in our population, and its influence on the utility of lactose metabolites as FIBs of milk intake. Similarly, the status of galactoside 2-alpha-L-fucosyltransferase 2 (FUT2) and galactoside 2-alpha-L-fucosyltransferase 3 (FUT3) enzymes, which determines the secretion of blood group antigens Lewis A trisaccharide and Blood Group H disaccharide that were previously proposed as candidate FIBs for milk intake [20], were also evaluated. We utilized whole-genome sequencing data that was performed for 737 NQplus participants, of which *n* = 110 overlapped with our validation sub-cohort. DNA was extracted from the blood samples of these participants using a Puregene 5Prime kit (Qiagen, Germantown, MD, USA) and sequenced using the Illumina OmniEspress chip (Illumina Inc., San Diego, CA, USA). We screened the single nucleotide polymorphisms (SNPs) data that was obtained through sequencing against a comprehensive list of SNPs associated with lactase persistence in the literature [54,55,56,57,58,59,60], which encompassed the common known functional SNPs rs4988235, rs182549, and rs41380347, as well as a number of rare variants (accession numbers: rs41456145, rs145946881, rs41525747, rs869051967, ss820496565, rs4988233, rs527991977, rs4954492, rs56348046, rs4954490, rs759157971). In addition, known SNPs for the *FUT2* (rs601338, rs1047781, rs281377, rs200157007) and *FUT3* (rs28362459, rs3745635, rs3894326, rs812936, rs778986) genes were also screened. From the screening, rs4988235 (13910C > T) was identified among the SNPs of the *LCT* gene and rs182549 (22018G > A) was identified for the upstream *MCM6* gene, both influencing lactase status, while rs601338 (G428A) was found for *FUT2* and rs778986 (C314T) for *FUT3*. Phenotypes for lactase (persistent and non-persistent) and FUT2/FUT3 status (secretors, non-secretors, and Lewis negative) were determined based on the SNPs.

### 4.7. Statistical Analysis

Participant characteristics are shown for the total population as well as stratified for sex as mean (SD), median (IQR) or *n* (%). Exploratory analyses were performed and metabolomics sample outliers, defined as observations clearly falling outside Hotelling’s T^2^ tolerance eclipse (95% confidence interval) in the principal component analysis (PCA) score plot, were identified and excluded (*n* = 23 LC-MS plasma, *n* = 2 LC-MS urine, and *n* = 24 GC-MS plasma).

Differences in levels of FIBs by quintiles of intake for dairy groups and dairy foods (for the total population, and sex-specific) were assessed by a Kruskal-Wallis test followed by a post-hoc Conover-Iman pairwise comparison test (*p* ≤ 0.05 as significance threshold). Spearman’s correlation coefficients (r_s_) were generated to analyse metabolite levels by continuous energy-adjusted g/d intakes (for the total population and by sex). Correlation coefficients of ≥0.50 were considered to be good, 0.20 to 0.49 as acceptable, and <0.20 as poor [61]. In addition, generalized linear models (GLM) with quasi-Poisson distribution were used to evaluate the performance of the candidate biomarkers in a ‘single-marker’ model in predicting the intake of different dairy foods. To avoid the use of negative values in the GLM, energy-adjusted intakes of dairy foods (g/d) were first offset by adding the minimum absolute intake value to all intake values of a food. Since the metabolite concentrations were compositional (i.e., they are expressed as relative abundance), we normalized metabolite concentrations prior to analysis using a centered log ratio (CLR) transformation [62,63,64]. CLR transformation was performed for the metabolite data using compositions R package (v2.0-0) [65].

To evaluate whether a ‘multi-marker’ panel consisting of a combination of FIBs performed better than single FIBs in predicting intakes, stepwise regression models (forwards and backwards) were generated for biomarkers per platform and per biosample for milk, cheese, and yoghurt. For dairy groups (total dairy, fermented dairy, non-fermented dairy, and their high- and low-fat variations), a combination of plasma C15:0 and C17:0 was investigated. Further, FIBs with significant spearman’s correlations r_s_ > 0 for intake of fermented dairy products (total, high-fat, and low-fat fermented dairy, cheese, yogurt) and r_s_ < 0 for intake of non-fermented dairy products (total, high-fat, and low-fat non-fermented dairy, milk) were further modelled using stepwise regression and a multi-marker approach to investigate which FIBs can help distinguish between fermented and non-fermented dairy intake. The best multi-marker models were selected based on the lowest quasi-Akaike Information Criterion (qAIC) value determined using the R package MuMIn (v1.43.17) [66] and presented in this paper. Multicollinearity of biomarkers were evaluated using the variance inflation factor (VIF), where VIF > 5 indicates potentially severe correlation between predictor variables, as confirmed/verified by pairwise correlations between biomarkers. In multi-marker models where high multicollinearity between several variables were observed, colinear variable(s) with the highest VIF were removed. Several categorical covariates were also added to adjust the regression models (0, 1): sex (male, female), BMI (normal weight < 25 kg/m^2^, overweight/obese ≥ 25/m^2^), and age (<55 years, ≥55 years (median split)).

For cross-validation of both the single-marker and multi-marker models, the dataset was randomly split into training (80%) and test datasets (20%). Spearman’s correlations between the actual and predicted values (r_ap_) were calculated to assess the strength and direction of the associations between these data, and performance accuracy of the models was further assessed by a coefficient of determination (R^2^) and mean absolute error (MAE), which was determined using the R package MLmetrics (v1.1.1) [67]. All statistics were performed in R (Version 3.6.3) [68]. For all models, the level of significance was set at *p* ≤ 0.05.

## 5. Conclusions

Multi-marker models factoring in several common physiological covariates was better able to capture the intakes of dairy products, including milk and cheese, over single-marker models. For yoghurt, prediction of intakes from both single- and multi-marker models were poor due to lack of specificity of the FIBs, or endogenous origin. Further evaluation of these FIBs as short-term biomarkers, quantification of these FIBs, and discovery of new fermentation biomarkers for dairy foods may help to improve estimates of dairy food intake and disentangle the health effects of dairy foods with different properties.

## Figures and Tables

**Table 1:** General characteristics of the participants ^a^.

	All (*n* = 246)	Men (*n* = 165)	Women (*n* = 81)	*p*-Value
Age, years	54.4 ± 12.5	55.9 ± 11.6	51.2 ± 13.6	0.01 **
BMI, kg/m^2^	25.9 ± 3.9	26.1 ± 3.6	25.4 ± 4.4	0.18
BMI-category, *n* (%)				0.010 **
<25 kg/m^2^	105 (42.7)	61 (37.0)	44 (54.3)	
≥25 kg/m^2^	141 (57.3)	104 (63.0)	37 (45.7)	
Waist circumference, cm	92.5 ± 11.6	95.8 ± 10.5	85.6 ± 10.7	<0.001 ***
Education, *n* (%)				0.38
Low	19 (7.7)	12 (7.3)	7 (8.8)	
Intermediate	77 (31.3)	49 (29.7)	28 (35.0)	
High	149 (60.6)	104 (63.0)	45 (56.2)	
Smoking status, *n* (%)				0.09
Never	119 (48.4)	71 (46.4)	48 (63.2)	
Former	85 (34.6)	65 (42.5)	20 (26.3)	
Current	25 (10.2)	17 (1.1)	8 (10.5)	
Disease history, *n* (%)				
Cancer	11 (4.5)	5 (3.0)	6 (7.4)	0.12
Diabetes	6 (2.4)	5 (3.0)	1 (1.2)	0.39
Heart attack	7 (2.8)	6 (3.6)	1 (1.2)	0.29
Hypertension	60 (24.4)	44 (26.7)	16 (19.8)	0.47
High cholesterol	52 (21.1)	38 (23.0)	14 (17.3)	0.58
Stroke	2 (0.8)	1 (0.6)	1 (1.2)	0.61
Diet during past month, *n* (%)				<0.001 ***
No	228 (92.7)	159 (96.4)	69 (85.2)	
Yes, always	9 (3.7)	1 (0.6)	8 (9.9)	
Yes, sometimes	9 (3.7)	5 (3.0)	4 (4.9)	
Lactase status, *n* (%)				1.00
Persistent	104 (94.5)	81 (94.2)	23 (95.8)	
Non-persistent	6 (5.5)	5 (5.8)	1 (4.2)	
FUT2/FUT3 status, *n* (%)				0.41
Secretor (Le a^−^b^+^)	87 (79.1)	69 (80.2)	18 (75.0)	
Non-secretor (Le a^+^b^−^)	19 (17.3)	13 (15.1)	6 (25.0)	
Lewis negative (Le a^−^b^−^)	4 (3.6)	4 (4.7)	0 (0)	

BMI, body mass index; FUT2, galactoside 2-alpha-L-fucosyltransferase 2; FUT3, galactoside 2-alpha-L-fucosyltransferase 3; SD, standard deviation. ** *p* ≤ 0.01, *** *p* ≤ 0.001. ^a^ Values are presented as mean ± SD, unless otherwise specified. Missing values: lactase, FUT2, and FUT3 status (*n* = 136), education (*n* = 1), smoking status (*n* = 17). Differences in characteristics between sexes were assessed using the t-test (for continuous variables), or chi-squared test (for categorical variables).

**Table 2:** Quintiles of intake for dairy groups and dairy foods (*n* = 246).

Food Group	Median Energy-Adjusted Intakes in g/d									
	n_c_	Q1 (*n* = 50)	n_c_	Q2 (*n* = 49)	n_c_	Q3 (*n* = 49)	n_c_	Q4 (*n* = 49)	n_c_	Q5 (*n* = 49)
Total dairy	50	98 (71, 129)	49	214 (197, 235)	49	304 (279, 323)	49	372 (355, 394)	49	527 (469, 616)
High-fat dairy	47	10 (6, 15)	49	24 (21, 28)	49	42 (35, 48)	49	73 (64, 81)	49	135 (109, 163)
Low-fat dairy	49	43 (25, 59)	49	148 (119, 173)	49	242 (224, 257)	49	317 (304, 340)	49	480 (404, 590)
Total fermented dairy	49	41 (24, 49)	49	90 (69, 109)	49	143 (134, 161)	49	224 (204, 237)	49	334 (291, 393)
High-fat fermented dairy	9	3 (−1, 4)	49	9 (7, 10)	49	17 (14, 19)	49	37 (30, 45)	49	82 (65, 117)
Low-fat fermented dairy	48	15 (7, 23)	49	50 (40, 62)	49	108 (99, 124)	49	195 (158, 210)	49	304 (269, 370)
Total non-fermented dairy	48	12 (4, 22)	49	54 (44, 63)	49	103 (91, 124)	49	179 (160, 207)	49	322 (282, 340)
High-fat non-fermented dairy	41	3 (1, 5)	49	10 (9, 12)	49	18 (16, 20)	49	31 (25, 35)	49	60 (48, 89)
Low-fat non-fermented dairy	0	−4 (−9, 5)	34	22 (14, 32)	49	69 (55, 89)	49	146 (127, 173)	49	293 (263, 373)
Cheese	46	8 (4, 12)	49	19 (17, 21)	49	27 (24, 29)	49	43 (39, 47)	49	67 (58, 90)
Yoghurt	16	0 (0, 5)	49	38 (22, 53)	49	83 (72, 96)	49	126 (105, 139)	49	193 (150, 212)
Milk	13	4 (−8, 14)	49	40 (29, 48)	49	87 (72, 108)	49	162 (144, 191)	49	303 (272, 371)

FFQ, food frequency questionnaire; n_c_, number of consumers. Values are reported as median (IQR), unless otherwise specified.

**Table 3:** Single-marker validation results for previously-identified candidate FIBs for milk.

Biomarker	Analytical Platform (Biosample) ^a^	Spearman’s Correlation Coefficient (r_s_)		Unadjusted GLM ^b^						Adjusted GLM ^b,c^					
				Coefficient	SE	*p*-Value	r_ap_	R^2^	MAE	Coefficient	SE	*p*-Value	r_ap_	R^2^	MAE
C15:0	GC-MS (P)	0.03	M: 0.05	(Int: 5.05) 0.04	(0.06) 0.12	(0.00 ***) 0.76	0.13	0.02	88.5	(Int: 5.24) 0.08	(0.12) 0.12	(0.00 ***) 0.49	0.09	0.01	89.0
			W: 0.00												
C17:0	GC-MS (P)	0.02	M: 0.06	(Int: 5.05) 0.03	(0.06) 0.14	(0.00 ***) 0.82	−0.12	0.01	88.5	(Int: 5.24) 0.08	(0.12) 0.14	(0.00 ***) 0.55	0.12	0.01	88.8
			W: 0.00												
Phenylalanine	LC-MS (P)	0.11	M: 0.03	(Int: 5.06) 0.08	(0.05) 0.05	(0.00 ***) 0.10	0.25	0.06	104.0	(Int: 5.29) 0.09	(0.11) 0.05	(0.00 ***) 0.07	0.47	0.22	99.8
			W: 0.32 **												
Tyrosine	LC-MS (P)	0.08	M: 0.01	(Int: 5.06) 0.08	(0.05) 0.05	(0.00 ***) 0.12	0.15	0.02	104.5	(Int: 5.29) 0.09	(0.11) 0.05	(0.00 ***) 0.06	0.53	0.28	100.5
			W: 0.25 *												
Tryptophan	LC-MS (P)	0.12	M: 0.06	(Int: 5.06) 0.11	(0.05) 0.06	(0.00 ***) 0.10	0.16	0.03	105.6	(Int: 5.28) 0.11	(0.11) 0.06	(0.00 ***) 0.09	0.38	0.14	101.7
			W: 0.25 *												
Indole-3-propionic acid	LC-MS (P)	0.04	M: −0.03	(Int: 5.07) 0.02	(0.05) 0.06	(0.00 ***) 0.68	0.05	0.00	106.4	(Int: 5.27) 0.02	(0.11) 0.06	(0.00 ***) 0.75	0.40	0.16	102.7
			W: 0.16												
Indole-3-acetic acid	LC-MS (P)	0.10	M: −0.01	(Int: 5.06) 0.09	(0.05) 0.07	(0.00 ***) 0.18	−0.08	0.01	106.3	(Int: 5.26) 0.08	(0.11) 0.07	(0.00 ***) 0.22	0.25	0.07	103.7
			W: 0.29 *												
Lactose	GC-MS (U)	0.16 *	M: 0.23 **	(Int: 5.12) 0.12	(0.05) 0.06	(0.00 ***) 0.05	0.16	0.03	91.8	**(Int: 5.30)** **0.13**	(**0.11**) **0.06**	(0.00 ***) 0.03 *	**0.20**	**0.04**	**92.7**
			W: 0.08												
	GC-MS (P)	−0.01	M: −0.05	(Int: 5.05) 0.06	(0.06) 0.09	(0.00 ***) 0.55	0.09	0.01	88.3	(Int: 5.23) 0.05	(0.12) 0.09	(0.00 ***) 0.59	0.10	0.01	88.8
			W: 0.11												
Galactose	GC-MS (U)	0.04	M: 0.11	(5.12) 0.04	(0.05) 0.03	(0.00 ***) 0.20	0.22	0.05	94.3	**(Int: 5.33)** **0.07**	(**0.11**) **0.03**	(0.00 ***) **0.04**	**0.21**	**0.04**	**93.0**
			W: 0.10												
	GC-MS (P)	−0.02	M: −0.02	(5.05) −0.11	(0.06) 0.27	(0.00 ***) 0.68	−0.08	0.01	89.4	(Int: 5.24) −0.12	(0.12) 0.27	(0.00 ***) 0.65	0.08	0.01	88.5
			W: −0.02												
Galactitol	GC-MS (U)	0.20 **	M: 0.23 **	**(Int: 5.12)** **0.21**	**(0.05)** **0.10**	(0.00 ***) **0.03**	**0.17**	**0.03**	**93.6**	**(Int: 5.28)** **0.20**	(**0.11**) **0.10**	(0.00 ***) **0.04**	**0.17**	**0.03**	**93.3**
			W: 0.07												
	GC-MS (P)	0.00	M: −0.02	(Int: 5.05) 0.01	(0.06) 0.12	(0.00 ***) 0.94	−0.13	0.02	88.6	(Int: 5.24) 0.06	(0.12) 0.12	(0.00 ***) 0.60	0.14	0.02	88.7
			W: 0.05												
Galactonate	LC-MS (U)	0.14	M: 0.01	(Int: 5.12) 0.04	(0.05) 0.05	(0.00 ***) 0.36	0.12	0.01	96.7	(Int: 5.29) 0.05	(0.11) 0.05	(0.00 ***) 0.30	0.15	0.02	96.7
			W: 0.22												
	GC-MS (U)	0.04	M: 0.08	(Int: 5.12) 0.02	(0.05) 0.07	(0.00 ***) 0.72	0.19	0.04	95.6	(Int: 5.30) 0.05	(0.11) 0.07	(0.00 ***) 0.43	0.17	0.03	96.0
			W: 0.03												
	GC-MS (P)	0.02	M: 0.04	(Int: 5.05) 0.07	(0.06) 0.08	(0.00 ***) 0.36	0.13	0.02	87.8	(Int: 5.22) 0.09	(0.12) 0.08	(0.00 ***) 0.27	0.13	0.02	87.7
			W: 0.02												
Blood group H disaccharide	LC-MS (P)	−0.07	M: −0.10	(Int: 5.07) −0.02	(0.05) 0.05	(0.00 ***) 0.62	0.09	0.01	106.0	(Int: 5.27) −0.02	(0.11) 0.05	(0.00 ***) 0.63	0.38	0.15	102.3
			W: −0.06												
	LC-MS (U)	−0.05	M: 0.05	(Int: 5.12) 0.00	(0.05) 0.05	(0.00 ***) 0.94	0.04	0.00	97.1	(Int: 5.30) 0.03	(0.11) 0.05	(0.00 ***) 0.60	0.12	0.02	97.0
			W: −0.10												
Lewis A trisaccharide	LC-MS (P)	0.07	M: −0.01	(Int: 5.07) 0.00	(0.05) 0.04	(0.00 ***) 0.97	−0.01	0.00	106.4	(Int: 5.27) 0.00	(0.11) 0.04	(0.00 ***) 0.93	0.30	0.09	102.5
			W: 0.26 *												
Hippurate	GC-MS (U)	−0.10	M: −0.02	(Int: 5.12) −0.04	(0.05) 0.11	(0.00 ***) 0.73	−0.15	0.02	95.7	(Int: 5.30) 0.01	(0.11) 0.11	(0.00 ***) 0.91	0.12	0.02	95.2
			W: −0.08												
Methionine	GC-MS (P)	0.01	M: 0.03	(Int: 5.05) 0.08	(0.06) 0.13	(0.00 ***) 0.53	0.00	0.00	88.1	(Int: 5.25) 0.13	(0.12) 0.13	(0.00 ***) 0.32	0.10	0.01	87.5
			W: 0.05												

C15:0, pentadecanoic; C17:0, heptadecanoic acid; FIB, food intake biomarker; GC-MS, gas chromatography mass spectrometry; GLM, generalized linear model; LC-MS, liquid chromatography mass spectrometry; M, men; MAE, mean absolute error; P, plasma; r_ap_, correlation between actual and predicted intake; SE, standard error; U, urine; W, women. Significant results are bolded: * *p* ≤ 0.05, ** *p* ≤ 0.01, *** *p* ≤ 0.001. ^a^ For the current study, biomarkers that were previously detected in serum were validated in plasma. For biomarkers that were previously detected using NMR, GC-MS was used as a substitution platform. A few biomarkers were not visible [asparagine (LC-MS plasma), taurine (LC-MS plasma), allantoin (GC-MS urine)] or not detected [galactono-1,5-lactone (GC-MS plasma and urine), galactonate (LC-MS plasma), gluconic acid and delta-gluconolactone (LC-MS plasma)] and were therefore not included in the current validation. ^b^ Intercept (Int) values for the models are provided in brackets. ^c^ Adjusted for age, sex, and BMI.

**Table 4:** Single-marker validation results for previously-identified candidate FIBs for cheese.

Biomarker	Analytical Platform (Biosample) ^a^	Spearman’s Correlation Coefficient (r_s_)		Unadjusted GLM ^b^						Adjusted GLM ^b,c^					
				Coefficient	SE	*p*-Value	r_ap_	R^2^	MAE	Coefficient	SE	*p*-Value	r_ap_	R^2^	MAE
C15:0	GC-MS (P)	0.12	M: 0.15	(Int: 3.91) 0.12	(0.03) 0.07	(0.00 ***) 0.10	0.21	0.04	15.5	(Int: 4.00) 0.12	(0.07) 0.07	(0.00 ***) 0.11	0.10	0.01	16.0
			W: 0.07												
C17:0	GC-MS (P)	0.08	M: 0.12	(Int: 3.91) 0.10	(0.03) 0.08	(0.00 ***) 0.23	0.19	0.04	15.8	(Int: 4.00) 0.10	(0.07) 0.08	(0.00 ***) 0.23	0.03	0.00	16.3
			W: 0.04												
3-Phenyllactic acid	GC-MS (U)	−0.11	M: 0.08	(Int: 3.97) 0.01	(0.03) 0.04	(0.00 ***) 0.78	−0.17	0.03	18.9	(Int: 4.099) 0.00	(0.07) 0.04	(0.00 ***) 0.98	0.16	0.02	18.5
			W: 0.08												
	GC-MS (P)	−0.05	M: 0.07	(Int: 3.91) −0.06	(0.03) 0.08	(0.00 ***) 0.45	−0.07	0.01	16.4	(Int: 3.99) −0.05	(0.07) 0.08	(0.00 ***) 0.58	−0.02	0.00	16.7
			W: −0.17												
3-Hydroxy-isobutyrate	GC-MS (P)	−0.04	M: −0.04	(Int: 3.91) 0.00	(0.03) 0.07	(0.00 ***) 0.99	−0.04	0.00	16.2	(Int: 3.99) 0.00	(0.07) 0.08	(0.00 ***) 0.95	0.03	0.00	16.6
			W: 0.01												
Phenylalanyl-proline	LC-MS (P)	0.05	M: 0.01	(Int: 3.88) 0.04	(0.04) 0.03	(0.00 ***) 0.17	−0.11	0.01	20.6	(Int: 4.06)0.05	(0.08) 0.03	(0.00 ***) 0.09	0.07	0.01	20.3
			W: 0.18												
	LC-MS (U)	−0.07	M: −0.08	(Int: 3.97) 0.00	(0.03) 0.08	(0.00 ***) 0.97	−0.12	0.01	22.5	(Int: 4.11) 0.04	(0.07) 0.08	(0.00 ***) 0.68	−0.06	0.00	22.7
			W: 0.01												
Indole-3-lactic acid	LC-MS (P)	0.06	M: 0.02	(Int: 3.89) 0.05	(0.04) 0.04	(0.00 ***) 0.25	−0.07	0.01	20.8	(Int: 4.05) 0.05	(0.08) 0.04	(0.00 ***) 0.24	0.07	0.01	20.5
			W: 0.18												
	LC-MS (U)	0.13	M: 0.20 *	(Int: 3.97) 0.18	(0.03) 0.11	(0.00 ***) 0.11	0.11	0.01	22.2	(Int: 4.08) 0.10	(0.08) 0.12	(0.00 ***) 0.40	0.04	0.00	22.6
			W: 0.13												
Proline	LC-MS (U)	−0.05	M: 0.03	(Int: 3.97) 0.06	(0.03) 0.07	(0.00 ***) 0.40	0.24	0.06	22.3	(Int: 4.10) 0.07	(0.07) 0.07	(0.00 ***) 0.32	0.04	0.00	22.6
			W: 0.05												
	GC-MS (P)	−0.16 *	M: −0.15	(Int: 3.9) −0.07	(0.03) 0.04	(0.00 ***) 0.07	0.05	0.00	17.1	(Int: 3.98) −0.07	(0.07) 0.04	(0.00 ***) 0.10	0.01	0.00	17.7
			W: −0.11												
Alanine	GC-MS (U)	0.12	M: 0.04	(Int: 3.97) 0.00	(0.03) 0.05	(0.00 ***) 0.96	−0.22	0.05	18.9	(Int: 4.10) 0.02	(0.07) 0.05	(0.00 ***) 0.73	0.07	0.00	18.5
			W: −0.14												
Pyroglutamate	GC-MS (U)	−0.01	M: −0.06	(Int: 3.97) −0.12	(0.03) 0.10	(0.00 ***) 0.24	0.11	0.01	18.4	(Int: 4.09) −0.08	(0.07) 0.10	(0.00 ***) 0.45	0.26	0.07	18.2
			W: −0.09												
Methionine	GC-MS (P)	−0.14	M: −0.10	(Int: 3.91) −0.13	(0.03) 0.08	(0.00 ***) 0.08	−0.02	0.00	17.3	(Int: 3.98) −0.13	(0.07) 0.08	(0.00 ***) 0.10	−0.08	0.01	17.8
			W: −0.16												
Leucine	GC-MS (P)	−0.11	M: −0.03	(Int: 3.91) −0.14	(0.03) 0.10	(0.00 ***) 0.15	−0.09	0.01	16.7	(Int: 3.98) −0.14	(0.07) 0.11	(0.00 ***) 0.19	−0.04	0.00	17.1
			W: −0.19												
Glutamic acid	GC-MS (P)	−0.04	M: 0.00	(Int: 3.91) −0.02	(0.03) 0.05	(0.00 ***) 0.77	−0.01	0.00	16.3	(Int: 3.99) −0.01	(0.07) 0.05	(0.00 ***) 0.81	−0.02	0.00	16.8
			W: −0.05												
Valine	GC-MS (P)	−0.12	M: −0.08	(Int: 3.91) −0.13	(0.03) 0.08	(0.00 ***) 0.13	−0.05	0.00	17.0	(Int: 3.98) −0.12	(0.07) 0.09	(0.00 ***) 0.16	−0.07	0.01	17.5
			W: −0.13												
Isoleucine	GC-MS (P)	−0.12	M: −0.06	(Int: 3.91) −0.14	(0.03) 0.08	(0.00 ***) 0.08	−0.10	0.01	17.2	(Int: 3.97) −0.13	(0.07) 0.08	(0.00 ***) 0.12	−0.07	0.00	17.5
			W: −0.20												

C15:0, pentadecanoic; C17:0, heptadecanoic acid; FIB, food intake biomarker; GC-MS, gas chromatography mass spectrometry; GLM, generalized linear model; LC-MS, liquid chromatography mass spectrometry; M, men; MAE, mean absolute error; P, plasma; r_ap_, correlation between actual and predicted intake; SE, standard error; U, urine; W, women. Significant results are bolded: * *p* ≤ 0.05, *** *p* ≤ 0.001. ^a^ For the current study, biomarkers that were previously detected in serum were validated in plasma. For biomarkers that were previously detected using NMR, GC-MS was used as a substitution platform. A few biomarkers were not visible [aminoadipic acid (LC-MS plasma and urine), citrulline (LC-MS plasma), valyl-threonine (LC-MS plasma)] and were therefore not included in the current validation. ^b^ Intercept (Int) values for the models are provided in brackets. ^c^ Adjusted for age, sex, and BMI.

**Table 5:** Single-marker validation results for previously-identified candidate FIBs for yoghurt.

Biomarker	Analytical Platform (Biosample) ^a^	Spearman’s Correlation Coefficient (r_s_)		Unadjusted GLM ^b^						Adjusted GLM ^b,c^					
				Coefficient	SE	*p*-Value	r_ap_	R^2^	MAE	Coefficient	SE	*p*-Value	r_ap_	R^2^	MAE
Proline	LC-MS (P)	0.01	M: 0.01	(Int: 4.53) −0.01	(0.06) 0.06	(0.00 ***) 0.89	0.13	0.02	68.0	(Int: 4.69) 0.01	(0.13) 0.06	(0.00 ***) 0.92	−0.12	0.02	68.5
			W: 0.17												
Indole-3-lactic acid	LC-MS (P)	0.03	M: 0.01	(Int: 4.53) 0.02	(0.06) 0.08	(0.00 ***) 0.80	−0.05	0.00	67.8	(Int: 4.68) 0.03	(0.13) 0.08	(0.00 ***) 0.73	−0.15	0.02	68.7
			W: 0.14												
Lysine	LC-MS (P)	0.02	M: −0.02	(Int: 4.53) 0.01	(0.06) 0.07	(0.00 ***) 0.89	0.08	0.01	67.8	(Int: 4.69) 0.02	(0.13) 0.07	(0.00 ***) 0.81	−0.16	0.03	68.5
			W: 0.20												
Threonine	LC-MS (P)	0.04	M: −0.01	(Int: 4.53) −0.01	(0.06) 0.06	(0.00 ***) 0.92	0.02	0.00	67.9	(Int: 4.68) −0.00	(0.13) 0.06	(0.00 ***) 0.97	−0.13	0.02	68.5
			W: 0.20												
Phenylalanine	LC-MS (P)	0.08	M: 0.07	(Int: 4.53) 0.03	(0.06) 0.06	(0.00 ***) 0.64	0.01	0.00	67.6	(Int: 4.69) 0.04	(0.13) 0.06	(0.00 ***) 0.53	−0.12	0.01	68.3
			W: 0.17												
Tyrosine	LC-MS (P)	0.12	M: 0.10	(Int: 4.52) 0.06	(0.06) 0.06	(0.00 ***) 0.29	−0.09	0.01	67.4	(Int: 4.70) 0.07	(0.13) 0.06	(0.00 ***) 0.21	−0.15	0.02	68.1
			W: 0.21												
Tryptophan	LC-MS (P)	0.03	M: 0.02	(Int: 4.53) 0.02	(0.06) 0.08	(0.00 ***) 0.83	−0.15	0.02	68.0	(Int: 4.69) 0.02	(0.13) 0.07	(0.00 ***) 0.75	−0.17	0.03	69.0
			W: 0.10												
Indole-3-acetaldehyde	LC-MS (P)	0.03	M: 0.00	(Int: 4.53) 0.03	(0.06) 0.09	(0.00 ***) 0.70	−0.17	0.03	67.7	(Int: 4.69) 0.05	(0.13) 0.09	(0.00 ***) 0.59	−0.15	0.02	68.4
			W: 0.15												

FIB, food intake biomarker; GLM, generalized linear model; LC-MS, liquid chromatography mass spectrometry; M, men; MAE, mean absolute error; P, plasma; r_ap_, correlation between actual and predicted intake; SE, standard error; W, women. Significant results are bolded: *** *p* ≤ 0.001. ^a^ For the current study, biomarkers that were previously detected in serum were validated in plasma. A few biomarkers were not visible [citrulline (LC-MS plasma) and asparagine (LC-MS plasma)] and were therefore not included in the current validation. ^b^ Intercept (Int) values for the models are provided in brackets. ^c^ Adjusted for age, sex, and BMI.

**Table 6:** Single-marker validation results for pentadecanoic acid (C15:0) and heptadecanoic acid (C17:0) by dairy group.

Biomarker	Analytical Platform (Biosample)	Spearman’s Correlation Coefficient (r_s_)		Unadjusted GLM ^a^						Adjusted GLM ^a,b^					
				Coefficient	SE	*p*-Value	r_ap_	R^2^	MAE	Coefficient	SE	*p*-Value	r_ap_	R^2^	MAE
**Total Dairy**															
C15:0	GC-MS (P)	0.17 *	M: 0.17	**(Int: 5.89)** **0.16**	**(0.03)** **0.07**	(0.00 ***) 0.02 *	**0.06**	**0.00**	**130.9**	**(Int: 6.08)** **0.17**	**(0.07)** **0.07**	(0.00 ***) 0.02 *	**0.31**	**0.10**	**125.4**
			W: 0.13												
C17:0	GC-MS (P)	0.12	M: 0.14	(Int: 5.89) 0.15	(0.03) 0.08	(0.00 ***) 0.07	−0.01	0.00	128.1	(Int: 6.08) 0.16	(0.07) 0.08	(0.00 ***) 0.05	0.37	0.14	122.7
			W: 0.12												
**High-Fat Dairy**															
C15:0	GC-MS (P)	−0.01	M: −0.04	(Int: 4.22) 0.15	(0.06) 0.12	(0.00 ***) 0.21	0.03	0.00	56.2	(Int: 4.30) 0.16	(0.12) 0.12	(0.00 ***) 0.20	0.10	0.01	56.2
			W: 0.09												
C17:0	GC-MS (P)	−0.06	M: −0.08	(Int: 4.22) 0.10	(0.06) 0.14	(0.00 ***) 0.49	−0.03	0.00	56.0	(Int: 4.30) 0.10	(0.12) 0.14	(0.00 ***) 0.50	−0.01	0.00	56.1
			W: 0.00												
**Low-Fat Dairy**															
C15:0	GC-MS (P)	0.16 *	M: 0.19 *	(Int: 5.68) 0.16	(0.04) 0.09	(0.00 ***) 0.06	0.07	0.01	139.5	**(Int: 5.89)** **0.17**	**(0.08)** **0.09**	(0.00 ***) 0.05 *	**0.26**	**0.07**	**136.6**
			W: 0.07												
C17:0	GC-MS (P)	0.13	M: 0.17*	(Int: 5.68) 0.16	(0.04) 0.10	(0.00 ***) 0.11	0.04	0.00	137.9	(Int: 5.89) 0.17	(0.08) 0.10	(0.00 ***) 0.09	0.32	0.10	133.8
			W: 0.07												
**Total Fermented Dairy**															
C15:0	GC-MS (P)	0.24 ***	M: 0.24 **	**(Int: 5.25)** **0.27**	**(0.04)** **0.09**	(0.00 ***) 0.00 *	**0.01**	**0.00**	**107.3**	**(Int: 5.43)** **0.25**	**(0.09)** **0.09**	(0.00 ***) 0.01 **	**0.09**	**0.01**	**106.7**
			W: 0.21												
C17:0	GC-MS (P)	0.19 **	M: 0.20 *	**(Int: 5.25)** **0.26**	**(0.04)** **0.11**	(0.00 ***) 0.01 *	−**0.04**	**0.00**	**105.3**	**(Int: 5.43)** **0.23**	**(0.09)** **0.11**	(0.00 ***) 0.03 *	**0.06**	**0.00**	**103.7**
			W: 0.18												
**High-fat Fermented Dairy**															
C15:0	GC-MS (P)	0.05	M: 0.07	(Int: 3.80) 0.20	(0.06) 0.13	(0.00 ***) 0.11	0.18	0.03	35.7	(Int: 3.85) 0.24	(0.13) 0.13	(0.00 ***) 0.06	0.14	0.02	35.9
			W: 0.04												
C17:0	GC-MS (P)	0.01	M: 0.06	(Int: 3.80) 0.14	(0.06) 0.15	(0.00 ***) 0.34	0.10	0.01	36.2	(Int: 3.85) 0.17	(0.13) 0.15	(0.00 ***) 0.26	0.02	0.00	36.4
			W: −0.07												
**Low-Fat Fermented Dairy**															
C15:0	GC-MS (P)	0.19 **	M: 0.19 *	**(Int: 4.98)** **0.29**	**(0.06)** **0.12**	(0.00 ***) 0.01 *	−**0.03**	**0.00**	**101.1**	**(Int: 5.20)** **0.25**	**(0.11)** **0.12**	(0.00 ***) 0.03 *	**0.04**	**0.00**	**103.6**
			W: 0.19												
C17:0	GC-MS (P)	0.16 *	M: 0.15	**(Int: 4.99)** **0.30**	**(0.06)** **0.14**	(0.00 ***) 0.03 *	−**0.05**	**0.00**	**97.7**	(Int: 5.20) 0.25	(0.11) 0.13	(0.00 ***) 0.06	0.04	0.00	99.0
			W: 0.19												
**Total Non-Fermented Dairy**															
C15:0	GC-MS (P)	0.03	M: 0.06	(Int: 5.14) 0.04	(0.05) 0.11	(0.00 ***) 0.74	0.12	0.01	87.3	(Int: 5.33) 0.08	(0.11) 0.11	(0.00 ***) 0.48	0.07	0.01	88.3
			W: 0.02												
C17:0	GC-MS (P)	0.02	M: 0.06	(Int: 5.14) 0.03	(0.05) 0.13	(0.00 ***) 0.84	−0.11	0.01	87.2	(Int: 5.33) 0.07	(0.11) 0.13	(0.00 ***) 0.57	0.09	0.01	88.0
			W: 0.01												
**High-Fat Non-Fermented Dairy**															
C15:0	GC-MS (P)	−0.09	M: −0.12	(Int: 3.71) 0.03	(0.06) 0.13	(0.00 ***) 0.83	−0.13	0.02	29.5	(Int: 3.79) 0.00	(0.13) 0.13	(0.00 ***) 1.00	−0.20	0.04	29.9
			W: −0.01												
C17:0	GC-MS (P)	−0.12	**M:** −0.19 *	(Int: 3.71) 0.01	(0.06) 0.15	(0.00 ***) 0.96	−0.09	0.01	29.4	(Int: 3.79) −0.02	(0.13) 0.15	(0.000 ***) 0.88	−0.18	0.03	29.7
			W: 0.00												
**Low-Fat Non-Fermented Dairy**															
C15:0	GC-MS (P)	0.03	M: 0.10	(Int: 4.99) 0.03	(0.06) 0.13	(0.00 ***) 0.79	0.15	0.02	97.1	(Int: 5.19) 0.09	(0.12) 0.13	(0.00 ***) 0.48	0.19	0.04	95.0
			W: −0.05												
C17:0	GC-MS (P)	0.03	M: 0.12	(Int: 4.99) 0.03	(0.06) 0.15	(0.00 ***) 0.85	−0.14	0.02	97.1	(Int: 5.19) 0.09	(0.12) 0.15	(0.00 ***) 0.55	0.21	0.04	95.0
			W: −0.07												

C15:0, pentadecanoic; C17:0, heptadecanoic acid; GC-MS, gas chromatography mass spectrometry; GLM, generalized linear model; M, men; MAE, mean absolute error; P, plasma; r_ap_, correlation between actual and predicted intake; SE, standard error; W, women. Significant results are bolded: *p ≤ 0.05, ** *p* ≤ 0.01, *** *p* ≤ 0.001. ^a^ Intercept (Int) values for the models are provided in brackets. ^b^ Adjusted for age, sex, and BMI.

## Data Availability

The data presented in this study can be found in the article and accompanying supplemental files. Raw data are available on request from the corresponding author.

## Supplementary Materials

The following are available online at https://www.mdpi.com/article/10.3390/metabo11060395/s1, Table S1: quintiles of intake for dairy groups and dairy foods in men (*n* = 165), Table S2: quintiles of intake for dairy groups and dairy foods in women (*n* = 81), Table S3: previously-identified candidate FIBs for milk, cheese and yogurt with their platforms and biosamples of detection, Table S4: multi-marker validation results for previously-identified candidate FIBs for milk, cheese, and yoghurt (unadjusted models), Table S5: multi-marker validation results for previously-identified candidate FIBs for milk, cheese, and yoghurt (adjusted models), Table S6: multi-marker validation results for pentadecanoic acid (C15:0) and heptadecanoic acid (C17:0) by dairy group, Table S7: significant spearman’s correlations for fermented and non-fermented dairy groups, Table S8: multi-marker validation results for FIBs differentiating between fermented and non-fermented dairy intake by dairy group, Table S9: classification of dairy foods in the NQplus food frequency questionnaire, Table S10: list of suppliers of analytical standards, Table S11: identification features of compounds analyzed by LC-MS, Table S12: identification features of compounds analyzed by GC-MS, Figure S1: differences in metabolite levels by sex-specific quintiles of milk intake, Figure S2: levels of lactose metabolites, Figure S3: levels of Lewis system-related oligosaccharides by secretion status, Figure S4: differences in metabolite levels by sex-specific quintiles of cheese intake, Figure S5: differences in plasma levels of tyrosine by sex-specific quintiles of yoghurt intake, Figure S6: differences in plasma levels of heptadecanoic acid by quintiles of total non-fermented dairy intake, Figure S7: plasma FIBs positively correlated with fermented dairy intake and negatively correlated with non-fermented dairy intake.
