# Supplementary material for: Evaluating the Robustness of Biomarkers of Dairy Food Intake in a Free-Living Population Using Single- and Multi-Marker Approaches
Source: Metabolites. 2021 Jun 17;11(6):395. doi: 10.3390/metabo11060395 (PMC8235731; doi:10.3390/metabo11060395)
Supplement: Supplementary file 1 [file metabolites-11-00395-s001.zip › metabolites-1228961-supplementary.pdf]

metabolites

MDPI

Supplemental Material

Evaluating the robustness of biomarkers of dairy food intake in a free-living population using single- and multi-marker approaches

Katherine J. Li[1,2], Kathryn J. Burton-Pimentel[2*], Elske M. Brouwer-Brolsma[1], Edith J.M. Feskens[1], Carola Blaser[2], René Badertscher[2], Reto Portmann[2], Guy Vergères[2]

[1] Division of Human Nutrition and Health, Department of Agrotechnology and Food Sciences, Wageningen University & Research, P.O. Box 17, 6700 AA Wageningen, The Netherlands; katherine.li@wur.nl (K.J.L.); elske.brouwer-brolsma@wur.nl (E.M.B-B.); edith.feskens@wur.nl (E.J.M.F.)

[2] Agroscope, Federal Office for Agriculture (FOAG), Federal Department of Economic Affairs, Education and Research (EAER), Schwarzenburgstrasse 161, CH-3003 Bern, Switzerland; kathryn.pimentel@agroscope.admin.ch (K.J.B-P.); carola.blaser@agroscope.admin.ch (C.B.); rene.badertscher@agroscope.admin.ch (R.B.); reto.portmann@agroscope.admin.ch (R.P.); guy.vergeres@agroscope.admin.ch (G.V.)

* Correspondence: kathryn.pimentel@agroscope.admin.ch, Tel.: +41 (0) 58 467 3187

Table S1. Quintiles of intake for dairy groups and dairy foods in men (n = 165).

**Food Group**	**Median Energy-Adjusted Intakes in g/day**									
	**n_c**	**Q1 (n = 33)**	**n_c**	**Q2 (n = 33)**	**n_c**	**Q3 (n = 33)**	**n_c**	**Q4 (n = 33)**	**n_c**	**Q5 (n = 33)**
Total dairy	33	109 (801, 132)	33	216 (188, 237)	33	289 (270, 316)	33	359 (349, 383)	33	514 (465, 613)
High-fat dairy	32	12 (7, 16)	33	26 (22, 29)	33	44 (37, 51)	33	74 (65, 84)	33	138 (113, 176)
Low-fat dairy	33	39 (15, 55)	33	140 (118, 167)	33	237 (215, 254)	33	313 (298, 327)	33	472 (390, 597)
Total fermented dairy	33	47 (24, 59)	33	94 (76, 114)	33	143 (134, 156)	33	226 (205, 242)	33	329 (293, 355)
High-fat fermented dairy	7	2 (-1, 5)	33	10 (8, 11)	33	18 (16, 21)	33	35 (28, 46)	33	83 (67, 108)
Low-fat fermented dairy	32	16 (6, 24)	33	53 (43, 64)	33	110 (102, 122)	33	197 (157, 213)	33	304 (268, 348)
Total non-fermented dairy	32	15 (6, 23)	33	53 (42, 64)	33	102 (94, 114)	33	173 (159, 201)	33	300 (274, 360)
High-fat non-fermented dairy	27	4 (1, 6)	33	11 (10, 13)	33	19 (16, 21)	33	36 (30, 42)	33	62 (51, 91)
Low-fat non-fermented dairy	0	-3 (-8, 2)	19	17 (14, 23)	33	65 (53, 82)	33	137 (122, 153)	33	284 (255, 355)
Cheese	32	8 (4, 12)	33	19 (17, 23)	33	29 (27, 32)	33	45 (40, 49)	33	68 (62, 94)
Yoghurt	9	0 (-3, 5)	33	41 (27, 52)	33	88 (73, 94)	33	133 (109, 138)	33	189 (151, 208)
Milk	11	4 (-5, 11)	33	39 (30, 49)	33	82 (73, 96)	33	157 (142, 191)	33	287 (259, 355)

FFQ, food frequency questionnaire; n_c, number of consumers. Values are reported as median (IQR), unless otherwise specified.

Table S2. Quintiles of intake for dairy groups and dairy foods in women (n = 81).

**Food Group**	**Median Energy-Adjusted Intakes in g/day**									
	**n_c**	**Q1 (n = 17)**	**n_c**	**Q2 (n = 16)**	**n_c**	**Q3 (n = 16)**	**n_c**	**Q4 (n = 16)**	**n_c**	**Q5 (n = 16)**
Total dairy	17	87 (67, 115)	16	228 (207, 241)	16	338 (316, 344)	16	388 (370, 408)	16	545 (496, 608)
High-fat dairy	15	5 (3, 11)	16	23 (20, 26)	16	36 (32, 47)	16	67 (62, 67)	16	126 (107, 150)
Low-fat dairy	16	5 (29, 72)	16	160 (143, 189)	16	258 (242, 265)	16	338 (322, 349)	16	521 (449, 577)
Total fermented dairy	16	31 (16, 36)	16	84 (57, 93)	16	156 (137, 170)	16	216 (198, 228)	16	366 (317, 424)
High-fat fermented dairy	2	0 (-2, 2)	16	8 (6, 10)	16	16 (14, 21)	16	36 (29, 41)	16	80 (58, 120)
Low-fat fermented dairy	16	14 (9, 23)	16	39 (34, 52)	16	109 (90, 123)	16	189 (166, 203)	16	302 (275, 397)
Total non-fermented dairy	16	17 (-8, 40)	16	56 (52, 73)	16	119 (109, 128)	16	190 (163, 219)	16	345 (311, 375)
High-fat non-fermented dairy	14	1 (-2, 3)	16	9 (7, 10)	16	14 (13, 17)	16	22 (19, 27)	16	55 (38, 77)
Low-fat non-fermented dairy	0	6 (-16, 12)	15	39 (36, 43)	16	102 (67, 112)	16	159 (141, 182)	16	334 (292, 366)
Cheese	14	9 (5, 12)	16	16 (15, 17)	16	22 (21, 30)	16	39 (35, 42)	16	70 (55, 80)
Yoghurt	7	9 (3, 12)	16	26 (21, 39)	16	71 (63, 84)	16	124 (105, 135)	16	191 (155, 290)
Milk	2	11 (-10, 20)	16	43 (40, 51)	16	105 (96, 116)	16	168 (151, 196)	16	334 (295, 362)

FFQ, food frequency questionnaire; n_c, number of consumers. Values are reported as median (IQR), unless otherwise specified.

Table S3. Previously-identified candidate FIBs for milk, cheese and yoghurt with their platforms and biosamples of detection.

Biomarker	Analytical Platform	Biosample[a]	Reference
**Milk**			
Pentadecanoic acid (C15:0)	GC-MS	Serum	[1]
Heptadecanoic acid (C17:0)	GC-MS	Serum	[1]
Phenylalanine	LC-MS	Serum	[2]
Asparagine	LC-MS	Serum	[2]
Tyrosine	LC-MS	Serum	[2]
Tryptophan	LC-MS	Serum	[2]
Taurine	LC-MS	Serum	[2]
Indole-3-propionic acid	LC-MS	Serum	[2]
Indole-3-acetic acid	LC-MS	Serum	[2]
Lactose	GC-MS	Urine	[3]
	GC-MS	Serum	[1]
	NMR	Urine	[3]
Galactose	GC-MS	Urine	[3]
	GC-MS	Serum	[1]
	NMR	Urine	[3]
Galactono-1,5-lactone	GC-MS	Urine	[3]
	GC-MS	Serum	[1]
Galacitol	GC-MS	Urine	[3]
	GC-MS	Serum	[1]
Galactonate	LC-MS	Serum	[4]
	LC-MS	Urine	[4]
	NMR	Urine	[3]
	GC-MS	Serum	[1]
Gluconic acid	LC-MS	Serum	[2]
Delta-Gluconolactone	LC-MS	Serum	[2]
Blood group H disaccharide	LC-MS	Serum	[4]
	LC-MS	Urine	[4]
Lewis A trisaccharide	LC-MS	Serum	[4]
Allantoin	NMR	Urine	[3]
Hippurate	NMR	Urine	[3]
Methionine	NMR	Serum	[1]
**Cheese**			
Pentadecanoic acid (C15:0)	GC-MS	Serum	[1]
Heptadecanoic acid (C17:0)	GC-MS	Serum	[1]
3-Phenyllactic acid	GC-MS	Urine	[3]
	GC-MS	Serum	[1]
3-Hydroxyisobutyrate	NMR	Serum	[1]
Aminoadipic acid	LC-MS	Serum	[4]
	LC-MS	Urine	[4]
Citrulline	LC-MS	Serum	[4]
Valyl-threonine	LC-MS	Serum	[4]
Phenylalanyl-proline	LC-MS	Serum	[4]

	LC-MS	Urine	[4]
Indole-3-lactic acid	LC-MS	Serum	[4]
	LC-MS	Urine	[4]
Proline	LC-MS	Serum	[4]
	NMR	Urine	[3]
	GC-MS	Serum	[1]
Alanine	GC-MS	Urine	[3]
	NMR	Urine	[3]
Pyroglutamate	NMR	Urine	[3]
Methionine	GC-MS	Serum	[1]
	NMR	Serum	[1]
Leucine	GC-MS	Serum	[1]
Glutamic acid	GC-MS	Serum	[1]
Valine+isoleucine	NMR	Serum	[1]
**Yoghurt**			
Proline	LC-MS	Serum	[2]
Indole-3-lactic acid	LC-MS	Serum	[2]
Citrulline	LC-MS	Serum	[2]
Lysine	LC-MS	Serum	[2]
Threonine	LC-MS	Serum	[2]
Phenylalanine	LC-MS	Serum	[2]
Asparagine	LC-MS	Serum	[2]
Tyrosine	LC-MS	Serum	[2]
Tryptophan	LC-MS	Serum	[2]
Indole-3-acetaldehyde	LC-MS	Serum	[2]

FIB, food intake biomarker; GC-MS, gas chromatography mass spectrometry; LC-MS, liquid chromatography mass spectrometry; NMR, nuclear magnetic resonance.

metabolites

MDPI

Table S4. Multi-marker validation results for previously-identified candidate FIBs for milk, cheese, and yoghurt (Unadjusted Models)[a]

Analytical Platform	Biosample	Biomarker[b]		qAIC	Coefficient	SE	t-value	*p*-value	r_{ap}	R^2	MAE
**Milk**											
GC-MS	(P)	C15:0		205.2	(Int: 5.05)<br>Galactonate: 0.07	(0.06)<br>0.08	(87.82)<br>0.92	(0.00***)<br>0.36	0.13	0.02	87.8
		C17:0									
		Lactose									
		Galactose									
		Galactitol									
		Methionine									
		Galactonate	x								
	(U)	Lactose		221.7	**(Int: 5.12)**<br>**Galactitol: 0.21**	**(0.05)**<br>**0.10**	**(103.53)**<br>**2.18**	**(0.00***)**<br>**0.03***	**0.17**	**0.03**	**93.6**
		Galactose									
		Galactitol	x								
		Hippurate									
		Galactonate									
LC-MS	(P)	Phenylalanine	x	210.0	(Int: 5.06)<br>Phenylalanine: 0.08	(0.05<br>0.05	(94.39)<br>1.64	(0.00***)<br>0.10	0.25	0.06	104.0
		Tyrosine									
		Tryptophan									
		Indole-3-propionic acid (IPA)									
		Indole-3-acetic acid									
		Lewis A disaccharide									
		Blood Group H trisaccharide									
	(U)	Blood Group H trisaccharide		218.9	(Int: 5.12)<br>Galactonate: 0.04	(0.05)<br>0.05	(102.30)<br>0.92	(0.00***)<br>0.36	0.12	0.01	96.7
		Galactonate	x								
**Cheese**											
GC-MS	(P)	C15:0	x (VIF=1.3)	294.4	**(Int: 3.91)**<br>**C15: 0.21**<br>**Isoleucine: -0.34**<br>**Glutamic acid: 0.12**	**(0.03)**<br>**0.07**<br>**0.11**<br>**0.07**	**(116.50)**<br>**2.78**<br>**-3.03**<br>**1.79**	**(0.00***)**<br>**0.01****<br>**0.00****<br>**0.08**	**0.16**	**0.03**	**16.8**
		C17:0									
		3-Phenyllactic acid									
		3-Hydroxyisobutyrate (HIB)									

Analytical Platform	Biosample	Biomarker[b]		qAIC	Coefficient	SE	t-value	*p*-value	r_{ap}	R^2	MAE
		Proline									
		Methionine									
		Leucine									
		Glutamic acid	x (VIF=1.9)								
		Valine									
		Isoleucine	x (VIF=2.2)								
	(U)	3-Phenyllactic acid		300.4	(Int: 3.97)<br>Pyroglutamate: -0.12	0.03<br>0.10	120.99<br>-1.18	(0.00***)<br>0.24	0.11	0.01	18.4
		Alanine									
		Pyroglutamate	x								
LC-MS	(P)	Indole-3-lactic acid (ILA)		286.2	(Int: 3.88)<br>Proline: 0.06	0.04<br>0.03	108.94<br>1.66	(0.00***)<br>0.10	0.06	0.00	20.2
		Proline	x								
		Phenylalanyl-proline									
	(U)	Indole-3-lactic acid	x	300.0	(Int: 3.97)<br>Indole-3-lactic acid: 0.18	0.03<br>0.11	120.23<br>1.62	(0.00***)<br>0.11	0.11	0.01	22.2
		Phenylalanyl-proline									
		Proline									
**Yoghurt**											
LC-MS	(P)	Proline		217.9	(Int: 4.52)<br>Threonine: -0.15<br>Tyrosine: 0.17	0.06<br>0.10<br>0.09	71.86<br>-1.51<br>1.81	(0.00***)<br>0.13<br>0.07	0.03	0.00	68.5
		Indole-3-lactic acid									
		Lysine									
		Threonine	x (VIF=2.6)								
		Phenylalanine									
		Tyrosine	x (VIF=2.6)								
		Tryptophan									
		Indole-3-acetaldehyde									

C15:0, pentadecanoic acid; C17:0, heptadecanoic acid; FIB, food intake biomarker; GC-MS, gas chromatography mass spectrometry; MAE, mean absolute error; NA, not applicable; P, plasma; qAIC, quasi-Akaike Information Criterion; r_{ap} = correlation between actual and predicted intake; SE, standard error; VIF, variance inflation factor. Significant results are bolded: *$p \leq 0.05$, ** $p \leq 0.01$, *** $p \leq 0.001$.

[a] Intercept (Int) values for the models are provided in brackets.

[b] Biomarkers and/or covariates included in the model are indicated with an 'x'.

Table S5. Multi-marker validation results for previously-identified candidate FIBs for milk, cheese, and yoghurt (adjusted models)[a]

Analytical Platform	Biosample	Biomarker[b]		Sex	BMI	Age	qAIC	Coefficient	SE	t-value	*p*-value	r_{ap}	R^2	MAE
**Milk**														
GC-MS	(P)	C15:0<br>C17:0<br>Lactose<br>Galactose<br>Galactitol<br>Methionine<br>Galactonate		x			206.4	(Int: 5.18)<br>Sex: -0.21	(0.09)<br>0.12	(57.48)<br>-1.81	(0.00***)<br>0.07	0.09	0.01	88.4
	(U)	Lactose<br>Galactose<br>Galactitol<br>Hippurate<br>Galactonate	 x (VIF=1.1)<br>x (VIF=1.0) 	x	x	x	224.2	**(Int: 5.31)**<br>**Galactose: 0.06**<br>**Galactitol: 0.18**<br>**Age: 0.17**<br>**Sex: -0.27**<br>**BMI: -0.19**	**(0.11)**<br>**0.03**<br>**0.10**<br>**0.11**<br>**0.11**<br>**0.10**	**(49.69)**<br>**1.83**<br>**1.83**<br>**1.58**<br>**-2.43**<br>**-1.81**	**(0.00***)**<br>**0.07**<br>**0.07**<br>**0.12**<br>**0.02***<br>**0.07**	**0.20**	**0.04**	**91.8**
LC-MS	(P)	Phenylalanine<br>Tyrosine<br>Tryptophan<br>Indole-3-propionic acid (IPA)<br>Indole-3-acetic acid<br>Lewis A disaccharide<br>Blood Group H trisaccharide	 <br>x (VIF=2.7)<br>x (VIF=2.7) 	x			212.4	**(Int: 5.23)**<br>**IPA: -0.15**<br>**Tryptophan: 0.24**<br>**Sex: -0.26**	**(0.09)**<br>**0.09**<br>**0.10**<br>**0.11**	**(60.18)**<br>**-1.70**<br>**2.41**<br>**-2.31**	**(0.00***)**<br>**0.09**<br>**0.02***<br>**0.02***	**0.25**	**0.06**	**102.8**
	(U)	Blood Group H trisaccharide<br>Galactonate		x			220.0	(Int: 5.25)<br>Sex: -0.20	(0.08)<br>0.10	63.29)<br>-1.93	(0.00***)<br>0.06	0.11	0.01	95.6
**Cheese**														
GC-MS	(P)	C15:0<br>C17:0<br>3-Phenyllactic acid<br>3-Hydroxyisobutyrate (HIB)	x (VIF=1.3) 				289.4	**(Int: 3.91)**<br>**C15: 0.21**<br>**Isoleucine: -0.34**	**(0.03)**<br>**0.08**<br>**0.11**<br>**0.07**	**(116.50)**<br>**2.78**<br>**-3.03**<br>**1.79**	**(0.00***)**<br>**0.01***<br>**0.00***<br>**0.08**	**0.16**	**0.03**	**16.8**

Analytical Platform	Biosample	Biomarker[b]		Sex	BMI	Age	qAIC	Coefficient	SE	t-value	*p*-value	r_{ap}	R^2	MAE
		Proline						**Glutamic acid: 0.12**						
		Methionine												
		Leucine												
		Glutamic acid	x (VIF=1.9)											
		Valine												
		Isoleucine	x (VIF=2.2)											
	(U)	3-Phenyllactic acid				x	301.9	**(Int: 4.01)**	**(0.04)**	**(103.50)**	**(0.00***)**	**0.27**	**0.07**	**18.4**
		Alanine						**Age: -0.14**	**0.07**	**-2.02**	**0.05***			
		Pyroglutamate												
LC-MS	(P)	Indole-3-lactic acid (ILA)		x	x		292.0	(Int: 4.03)	(0.07	(56.31)	(0.00***)	0.10	0.01	20.3
		Proline	x					Proline: 0.07	0.03	1.93	0.06			
		Phenylalanyl-proline						Sex: -0.15	0.08	-1.89	0.06			
								BMI: -0.13	0.07	-1.77	0.08			
	(U)	Indole-3-lactic acid				x	301.4	**(Int: 4.01)**	**(0.04)**	**(102.74)**	**(0.00***)**	**0.16**	**0.02**	**21.9**
		Phenylalanyl-proline						**Age: -0.14**	**0.07**	**-1.98**	**0.05***			
		Proline												
**Yoghurt**														
LC-MS	(P)	Proline		x			221.7	**(Int: 4.67)**	**(0.10)**	**(45.61)**	**(0.00***)**	**0.03**	**0.00**	**68.4**
		Indole-3-lactic acid						**Threonine: -0.18**	**0.10**	**-1.80**	**0.07**			
		Lysine						**Tyrosine: 0.20**	**0.09**	**2.16**	**0.03***			
		Threonine	x (VIF=2.7)					**Sex: -0.22**	**0.13**	**-1.72**	**0.09**			
		Phenylalanine												
		Tyrosine	x (VIF=2.8)											
		Tryptophan												
		Indole-3-acetaldehyde												

BMI, body mass index; C15:0, pentadecanoic acid; C17:0, heptadecanoic acid; FIB, food intake biomarker; GC-MS, gas chromatography mass spectrometry; MAE, mean absolute error; NA, not applicable; P, plasma; aAIC, quasi-Akaike Information Criterion; rap = correlation between actual and predicted intake; SE, standard error; VIF, variance inflation factor. Significant results are bolded: *$p \leq 0.05$, ** $p \leq 0.01$, *** $p \leq 0.001$.

[a] Intercept (Int) values for the models are provided in brackets.

[b] Biomarkers and/or covariates included in the model are indicated with an ‘x’.

Table S6. Multi-marker validation results for pentadecanoic acid (C15:0) and heptadecanoic acid (C17:0) by dairy group

	Analytical Platform (Biosample)	Biomarker		Sex	BMI	Age	qAIC	Coefficient	SE	t-value	*p*-value	r_{ap}	R^2	MAE
**Total Dairy**														
Unadjusted[a]	GC-MS (P)	C15:0<br>C17:0	x	NA	NA	NA	227.3	**(Int: 5.89)<br>C15: 0.16**	**(0.03)<br>0.07**	**(176.558)<br>2.318**	**(0.000***)<br>0.021***	**0.06**	**0.00**	**130.9**
Adjusted[a]	GC-MS (P)	C15:0<br>C17:0	x	x	x		227.9	**(Int: 6.05)<br>C15: 0.18<br>Sex: -0.16<br>BMI: -0.13**	**(0.06)<br>0.07<br>0.07<br>0.07**	**(94.379)<br>2.649<br>-2.348<br>-1.867**	**(0.000***)<br>0.009*<br>0.020*<br>0.063**	**0.31**	**0.10**	**122.5**
**High-fat Dairy**														
Unadjusted[a]	GC-MS (P)	C15:0<br>C17:0	x	NA	NA	NA	207.2	(Int: 4.22)<br>C15: 0.15	(0.06)<br>0.12	(73.557)<br>1.255	(0.000***)<br>0.211	0.03	0.00	56.2
Adjusted[a]	GC-MS (P)	C15:0<br>C17:0	x				205.2	(Int: 4.22)<br>C15: 0.15	(0.06)<br>0.12	(73.557)<br>1.255	(0.000***)<br>0.211	0.03	0.00	56.2
**Low-fat Dairy**														
Unadjusted[a]	GC-MS (P)	C15:0<br>C17:0	x	NA	NA	NA	222.5	(Int: 5.68)<br>C15: 0.16	(0.04)<br>0.09	(137.717)<br>1.905	(0.000***)<br>0.058	0.07	0.01	139.5
Adjusted[a]	GC-MS (P)	C15:0<br>C17:0	x	x	x		224.6	**(Int: 5.86)<br>C15: 0.19<br>Sex: -0.19<br>BMI: -0.13**	**(0.08)<br>0.09<br>0.09<br>0.08**	**(74.310)<br>2.201<br>-2.208<br>-1.564**	**(0.000***)<br>0.029*<br>0.028*<br>0.119**	**0.25**	**0.06**	**137.7**
**Total Fermented Dairy**														
Unadjusted[a]	GC-MS (P)	C15:0<br>C17:0	x	NA	NA	NA	215.0	**(Int: 5.25)<br>C15: 0.27**	**(0.04)<br>0.09**	**(121.322)<br>3.022**	**(0.000***)<br>0.003****	**0.01**	**0.00**	**107.3**
Adjusted[a]	GC-MS (P)	C15:0<br>C17:0	x	x			215.7	**(Int: 5.32)<br>C15: 0.23<br>Age: -0.24**	**(0.05)<br>0.09<br>0.10**	**(105.187)<br>2.586<br>-2.418**	**(0.000***)<br>0.010*<br>0.017***	**0.07**	**0.01**	**107.3**
**High-fat Fermented Dairy**														
Unadjusted[a]	GC-MS (P)	C15:0<br>C17:0	x	NA	NA	NA	198.9	(Int: 3.80)<br>C15: 0.20	(0.06)<br>0.13	(61.54)<br>1.59	(0.00***)<br>0.11	0.18	0.03	35.7
Adjusted[a]	GC-MS (P)	C15:0	X (VIF = 9.0)				203.9	(Int: 3.80)	(0.06)	(61.50)	(0.00***)	0.22	0.05	35.6

	Analytical Platform (Biosample)	Biomarker		Sex	BMI	Age	qAIC	Coefficient	SE	t-value	*p*-value	r_{ap}	R^2	MAE
		C17:0	x (VIF = 9.0)					C15: 0.68	0.34	1.97	0.05*			
								C17: -0.59	0.40	-1.47	0.14			
**Low-fat Fermented Dairy**														
Unadjusted[a]	GC-MS (P)	C15:0	x	NA	NA	NA	207.9	**(Int: 4.98)**	**(0.06)**	**(88.22)**	**(0.00***)**	**-0.03**	**0.00**	**101.1**
		C17:0						**C15: 0.29**	**0.12**	**2.50**	**0.01***			
Adjusted[a]	GC-MS (P)	C15:0	x			x	211.4	**(Int: 5.09)**	**(0.06)**	**(79.17)**	**(0.00***)**	**0.03**	**0.00**	**103.6**
		C17:0						**C15: 0.23**	**0.12**	**2.03**	**0.04***			
								**Age: -0.36**	**0.13**	**-2.77**	**0.01***			
**Total Non-fermented Dairy**														
Unadjusted[a]	GC-MS (P)	C15:0	x	NA	NA	NA	211.2	(Int: 5.14)	(0.05)	(97.24)	(0.00***)	0.12	0.01	87.3
		C17:0						C15: 0.04	0.11	0.33	0.74			
Adjusted[a]	GC-MS (P)	C15:0	x	x	x		212.1	**(Int: 5.35)**	**(0.10)**	**(53.74)**	**(0.00***)**	**0.09**	**0.01**	**87.2**
		C17:0						**BMI: -0.16**	**0.11**	**-1.47**	**0.14**			
								**Sex: -0.23**	**0.11**	**-2.14**	**0.03***			
**High-fat Non-fermented Dairy**														
Unadjusted[a]	GC-MS (P)	C15:0	x	NA	NA	NA	179.8	(Int: 3.71)	(0.06)	(60.83)	(0.00***)	-0.13	0.02	29.5
		C17:0						C15: 0.03	0.13	0.22	0.83			
Adjusted[a]	GC-MS (P)	C15:0				x	181.5	(Int: 3.77)	(0.07)	(53.18)	(0.00***)	0.14	0.02	29.1
		C17:0						Age: -0.19	0.13	-1.44	0.15			
**Low-fat Non-fermented Dairy**														
Unadjusted[a]	GC-MS (P)	C15:0	x	NA	NA	NA	203.1	(Int: 4.99)	(0.06)	(81.55)	(0.00***)	0.15	0.02	97.1
		C17:0						C15: 0.03	0.13	0.27	0.79			
Adjusted[a]	GC-MS (P)	C15:0		x	x		204.1	**(Int: 5.23)**	**(0.11)**	**(45.73)**	**(0.00***)**	**0.16**	**0.02**	**95.4**
		C17:0						**BMI: -0.18**	**0.13**	**-1.41**	**0.16**			
								**Sex: -0.27**	**0.13**	**-2.12**	**0.04***			

BMI, body mass index; C15:0, pentadecanoic acid; C17:0, heptadecanoic acid; GC-MS, gas chromatography mass spectrometry; MAE, mean absolute error; NA, not applicable; P, plasma; aAIC, quasi-Akaike Information Criterion; rap = correlation between actual and predicted intake; SE, standard error; VIF, variance inflation factor. Significant results are bolded: *$p \leq 0.05$, ** $p \leq 0.01$, *** $p \leq 0.001$.

[a] Intercept (Int) values for the models are provided in brackets.

[b] Biomarkers and/or covariates included in the model are indicated with an 'x'.

Table S7. Significant Spearman's Correlations for Fermented and Non-Fermented Dairy Groups

Platform (biosample)	Dairy Food/Group	Compound	Spearman's correlation coefficient (r_s)	*p*-value
GC-MS (P)	Cheese (fermented)	Proline	-0.160	0.022
GC-MS (P)	High-fat fermented dairy	Galactitol	-0.146	0.038
GC-MS (P)	High-fat fermented dairy	Galactonate	-0.158	0.024
GC-MS (P)	High-fat fermented dairy	Isoleucine	-0.159	0.023
GC-MS (P)	High-fat fermented dairy	Leucine	-0.152	0.030
GC-MS (P)	High-fat fermented dairy	Methionine	-0.160	0.022
GC-MS (P)	High-fat fermented dairy	Proline	-0.208	0.003
GC-MS (P)	High-fat fermented dairy	Valine	-0.178	0.011
GC-MS (P)	High-fat fermented dairy	3-Hydroxyisobutyrate	-0.185	0.008
GC-MS (P)	High-fat fermented dairy	3-Phenyllactic acid	-0.164	0.019
GC-MS (P)	Low-fat fermented dairy	Galactitol	0.145	0.038
GC-MS (P)	Low-fat fermented dairy	Galactonate	0.193	0.006
GC-MS (P)	Low-fat fermented dairy	Heptadecanoic acid (C17:0)	0.155	0.027
GC-MS (P)	Low-fat fermented dairy	Lactose	0.207	0.003
GC-MS (P)	Low-fat fermented dairy	Pentadecanoic acid (C15:0)	0.193	0.006
GC-MS (P)	Low-fat fermented dairy	Valine	0.141	0.044
GC-MS (P)	Low-fat fermented dairy	3-Hydroxyisobutyrate	0.229	0.001
GC-MS (P)	High-fat non-fermented dairy	Galactonate	-0.163	0.020
GC-MS (P)	High-fat non-fermented dairy	Glutamic acid	-0.162	0.021
GC-MS (P)	High-fat non-fermented dairy	Isoleucine	-0.269	0.000
GC-MS (P)	High-fat non-fermented dairy	Lactose	-0.145	0.039
GC-MS (P)	High-fat non-fermented dairy	Leucine	-0.152	0.030
GC-MS (P)	High-fat non-fermented dairy	Methionine	-0.236	0.001
GC-MS (P)	High-fat non-fermented dairy	Proline	-0.27	0.000
GC-MS (P)	High-fat non-fermented dairy	Valine	-0.279	0.000
GC-MS (P)	High-fat non-fermented dairy	3-Hydroxyisobutyrate	-0.244	0.000
GC-MS (P)	High-fat non-fermented dairy	3-Phenyllactic acid	-0.198	0.005
GC-MS (P)	Total fermented dairy	Galactitol	0.145	0.039
GC-MS (P)	Total fermented dairy	Galactonate	0.182	0.009

**Platform (biosample)**	**Dairy Food/Group**	**Compound**	**Spearman's correlation coefficient (r_s)**	***p*-value**
GC-MS (P)	Total fermented dairy	Heptadecanoic acid (C17:0)	0.187	0.007
GC-MS (P)	Total fermented dairy	Lactose	0.207	0.003
GC-MS (P)	Total fermented dairy	Pentadecanoic acid (C15:0)	0.236	0.001
GC-MS (P)	Total fermented dairy	3-Hydroxyisobutyrate	0.204	0.004
GC-MS (P)	Yoghurt (fermented)	Galactitol	0.146	0.037
GC-MS (P)	Yoghurt (fermented)	Galactonate	0.15	0.033
GC-MS (P)	Yoghurt (fermented)	Pentadecanoic acid (C15:0)	0.165	0.019
GC-MS (U)	Cheese (fermented)	Galactitol	0.221	0.001
GC-MS (U)	Cheese (fermented)	Lactose	0.187	0.006
GC-MS (U)	High-fat non-fermented dairy	Galactonate	0.227	0.001
GC-MS (U)	Low-fat fermented dairy	Galactonate	0.182	0.008
GC-MS (U)	Low-fat fermented dairy	3-Phenyllactic acid	0.223	0.001
GC-MS (U)	Low-fat non-fermented dairy	Galactitol	0.191	0.005
GC-MS (U)	Low-fat non-fermented dairy	Lactose	0.168	0.013
GC-MS (U)	Milk (non-fermented)	Alanine	0.135	0.048
GC-MS (U)	Milk (non-fermented)	Galactitol	0.199	0.003
GC-MS (U)	Milk (non-fermented)	Lactose	0.161	0.018
GC-MS (U)	Total fermented dairy	Galactonate	0.189	0.005
GC-MS (U)	Total fermented dairy	3-Phenyllactic acid	0.188	0.006
GC-MS (U)	Yoghurt (fermented)	Hippurate	0.141	0.038
LC-MS (P)	High-fat non-fermented dairy	Indole-3-acetaldehyde	0.174	0.012
LC-MS (P)	High-fat non-fermented dairy	Tryptophan	0.161	0.021
LC-MS (P)	High-fat non-fermented dairy	Phenylalanine	0.137	0.049
LC-MS (P)	Low-fat fermented dairy	Blood group H disaccharide	0.244	0.000
LC-MS (P)	Milk (non-fermented)	Threonine	0.144	0.039
LC-MS (P)	Total fermented dairy	Blood group H disaccharide	0.256	0.000
LC-MS (P)	Total fermented dairy	Tyrosine	0.158	0.024
LC-MS (P)	Total non-fermented dairy	Indole-3-acetaldehyde	0.153	0.029
LC-MS (P)	Total non-fermented dairy	Tryptophan	0.144	0.039
LC-MS (P)	Total non-fermented dairy	Lysine	0.138	0.048
LC-MS (P)	Total non-fermented dairy	Threonine	0.16	0.022

**Platform (biosample)**	**Dairy Food/Group**	**Compound**	**Spearman's correlation coefficient (r_s)**	***p*-value**
LC-MS (P)	Yoghurt (fermented)	Blood group H disaccharide	0.203	0.003
LC-MS (U)	Total non-fermented dairy	Galactonate	0.149	0.049

C15:0, pentadecanoic acid; C17:0, heptadecanoic acid; GC-MS, gas chromatography mass spectrometry; LC-MS, liquid chromatography mass spectrometry; P, plasma; U, urine. Significant indicated as: *$p \leq 0.05$, ** $p \leq 0.01$, *** $p \leq 0.001$.

Table S8. Multi-marker validation results for FIBs differentiating between fermented and non-fermented dairy intake by dairy group

	Analytical Platform (Biosample)	Biomarker	VIF	Coefficient	SE	t-value	*p*-value	r_{ap}	R^2	MAE
**Total dairy**										
Unadjusted[a]	GC-MS (P)	**(Int)**	**NA**	**(5.87)**	**(0.04)**	**(156.44)**	**(0.00***)**	**0.24**	**0.06**	**129.2**
		**C15:0**	**1.4**	**0.19**	**0.10**	**1.98**	**0.05***			
		**Lactose**	**1.4**	**0.18**	**0.06**	**3.04**	**0.00****			
		**Isoleucine**	**2.6**	**-0.12**	**0.14**	**-0.88**	**0.38**			
		**Proline**	**2.4**	**-0.03**	**0.07**	**-0.43**	**0.67**			
		**Galactonate**	**1.6**	**0.05**	**0.06**	**0.84**	**0.40**			
Adjusted[a]	GC-MS (P)	**(Int)**	**NA**	**(5.91)**	**(0.05)**	**(120.92)**	**(0.00***)**	**0.25**	**0.06**	**128.4**
		**C15:0**	**1.4**	**0.19**	**0.10**	**1.99**	**0.05***			
		**Lactose**	**1.4**	**0.19**	**0.06**	**3.20**	**0.00****			
		**BMI**	**1.1**	**-0.10**	**0.08**	**-1.27**	**0.21**			
		**Galactonate**	**1.7**	**0.06**	**0.06**	**1.02**	**0.31**			
		**Glutamic acid**	**3.3**	**-0.15**	**0.10**	**-1.47**	**0.14**			
		**Isoleucine**	**2.7**	**-0.08**	**0.14**	**-0.58**	**0.56**			
		**Proline**	**3.4**	**0.04**	**0.08**	**0.46**	**0.65**			
**High-fat dairy**										
Unadjusted[a]	GC-MS (P)	**(Int)**	**NA**	**(4.12)**	**(0.06)**	**(68.33)**	**(0.00***)**	**0.50**	**0.25**	**52.0**
		**C15:0**	**1.6**	**0.29**	**0.16**	**1.76**	**0.08**			
		**Glutamic acid**	**3.2**	**0.16**	**0.15**	**1.05**	**0.30**			
		**Proline**	**3.0**	**-0.21**	**0.12**	**-1.75**	**0.08**			
		**3-Hydroxyisobutyrate**	**2.4**	**-0.41**	**0.20**	**-2.04**	**0.04***			
Adjusted[a]	GC-MS (P)	**(Int)**	**NA**	**(4.12)**	**(0.06)**	**(68.33)**	**(0.00***)**	**0.50**	**0.25**	**52.0**
		**C15:0**	**1.6**	**0.29**	**0.16**	**1.76**	**0.08**			
		**3-Hydroxyisobutyrate**	**2.4**	**-0.41**	**0.20**	**-2.04**	**0.04***			
		**Glutamic acid**	**3.2**	**0.16**	**0.15**	**1.05**	**0.30**			
		**Proline**	**3.0**	**-0.21**	**0.12**	**-1.75**	**0.08**			
**Low-fat dairy**										
Unadjusted[a]	GC-MS (P)	**(Int)**	**NA**	**(5.67)**	**(0.04)**	**(125.96)**	**(0.00***)**	**0.27**	**0.07**	**131.4**
		**Galactonate**	**1.7**	**0.09**	**0.07**	**1.21**	**0.23**			

		**Lactose**	**1.4**	**0.21**	**0.07**	**3.21**	**0.00****			
		**Glutamic acid**	**3.0**	**-0.16**	**0.11**	**-1.40**	**0.16**			
		**Isoleucine**	**2.1**	**0.10**	**0.15**	**0.67**	**0.51**			
		**Proline**	**3.0**	**0.02**	**0.09**	**0.17**	**0.87**			
Adjusted[a]	GC-MS (P)	**(Int)**	**NA**	**(5.71)**	**(0.06)**	**(97.98)**	**(0.00***)**	**0.31**	**0.10**	**130.0**
		**Galactonate**	**1.7**	**0.09**	**0.07**	**1.24**	**0.22**			
		**Lactose**	**1.4**	**0.22**	**0.07**	**3.29**	**0.00****			
		**BMI**	**1.1**	**-0.10**	**0.10**	**-1.09**	**0.28**			
		**Glutamic acid**	**3.3**	**-0.20**	**0.12**	**-1.66**	**0.10**			
		**Isoleucine**	**2.1**	**0.09**	**0.15**	**0.62**	**0.53**			
		**Proline**	**3.0**	**0.03**	**0.09**	**0.34**	**0.73**			
**Total fermented dairy**										
Unadjusted[a]	GC-MS (P)	**(Int)**	**NA**	**(5.19)**	**(0.05)**	**(112.93)**	**(0.00***)**	**0.22**	**0.05**	**100.7**
		**Galactonate**	**1.6**	**0.07**	**0.07**	**0.95**	**0.34**			
		**Glutamic acid**	**2.4**	**-0.09**	**0.10**	**-0.87**	**0.38**			
		**Lactose**	**1.4**	**0.30**	**0.06**	**4.77**	**0.00*****			
		**C15:0**	**1.4**	**0.27**	**0.12**	**2.30**	**0.02***			
		**Methionine**	**3.0**	**-0.25**	**0.17**	**-1.48**	**0.14**			
		**3-Hydroxyisobutyrate**	**2.9**	**0.20**	**0.16**	**1.28**	**0.20**			
Adjusted[a]	GC-MS (P)	**(Int)**	**NA**	**(5.27)**	**(0.05)**	**(98.90)**	**(0.00***)**	**0.20**	**0.04**	**100.7**
		**C15:0**	**1.5**	**0.22**	**0.12**	**1.83**	**0.07**			
		**Lactose**	**1.4**	**0.28**	**0.06**	**4.59**	**0.00*****			
		**Age**	**1.1**	**-0.26**	**0.10**	**-2.52**	**0.01***			
		**Galactonate**	**1.7**	**0.03**	**0.07**	**0.47**	**0.64**			
		**Glutamic acid**	**2.5**	**-0.12**	**0.10**	**-1.17**	**0.24**			
		**Methionine**	**3.1**	**-0.21**	**0.17**	**-1.22**	**0.23**			
		**3-Hydroxyisobutyrate**	**2.9**	**0.25**	**0.16**	**1.57**	**0.12**			
**High-fat fermented dairy**										
Unadjusted[a]	GC-MS (P)	(Int)	NA	(3.72)	(0.07)	(57.27)	(0.00***)	0.37	0.14	34.6
		C15:0	1.6	0.31	0.18	1.77	0.08			
		3-Hydroxyisobutyrate	2.4	-0.41	0.22	-1.87	0.06			
		Glutamic acid	3.1	0.22	0.17	1.31	0.19			

		Proline	3.0	-0.14	0.13	-1.07	0.29			
Adjusted[a]	GC-MS (P)	**(Int)**	**NA**	**(3.80)**	**(0.08)**	**(46.43)**	**(0.00***)**	**0.40**	**0.16**	**35.1**
		**C15:0**	**1.3**	**0.34**	**0.16**	**2.18**	**0.03***			
		**3-Hydroxyisobutyrate**	**1.3**	**-0.40**	**0.16**	**-2.50**	**0.01***			
		**BMI**	**1.1**	**-0.19**	**0.14**	**-1.40**	**0.16**			
**Low-fat fermented dairy**										
Unadjusted[a]	GC-MS (P)	**(Int)**	**NA**	**(4.91)**	**(0.06)**	**(80.62)**	**(0.00***)**	**0.25**	**0.06**	**93.1**
		**C15:0**	**1.4**	**0.26**	**0.15**	**1.65**	**0.10**			
		**Lactose**	**1.4**	**0.34**	**0.08**	**4.53**	**0.00*****			
		**3-Hydroxyisobutyrate**	**2.8**	**0.37**	**0.21**	**1.79**	**0.08**			
		**Galactonate**	**1.6**	**0.11**	**0.09**	**1.21**	**0.23**			
		**Glutamic acid**	**2.4**	**-0.15**	**0.13**	**-1.17**	**0.25**			
		**Methionine**	**3.0**	**-0.27**	**0.22**	**-1.23**	**0.22**			
Adjusted[a]	GC-MS (P)	**(Int)**	**NA**	**(5.03)**	**(0.07)**	**(72.85)**	**(0.00***)**	**0.21**	**0.04**	**94.9**
		**C15:0**	**1.5**	**0.17**	**0.15**	**1.07**	**0.29**			
		**Lactose**	**1.4**	**0.32**	**0.07**	**4.31**	**0.00*****			
		**3-Hydroxyisobutyrate**	**3.3**	**0.44**	**0.22**	**2.05**	**0.04***			
		**Age**	**1.1**	**-0.40**	**0.14**	**-2.83**	**0.01****			
		**Galactonate**	**1.7**	**0.07**	**0.09**	**0.69**	**0.49**			
		**Glutamic acid**	**2.4**	**-0.19**	**0.13**	**-1.53**	**0.13**			
		**Leucine**	**2.4**	**-0.04**	**0.24**	**-0.16**	**0.87**			
		**Methionine**	**3.5**	**-0.19**	**0.23**	**-0.82**	**0.41**			
**Total non-fermented dairy**										
Unadjusted[a]	GC-MS (P)	(Int)	NA	(5.14)	(0.06)	(85.34)	(0.00***)	0.00	0.00	89.0
		3-Hydroxyisobutyrate	1.5	-0.25	0.16	-1.61	0.11			
		Galactonate	1.5	0.10	0.10	1.03	0.30			
		Proline	1.7	-0.01	0.10	-0.13	0.90			
Adjusted[a]	GC-MS (P)	**(Int)**	**NA**	**(5.44)**	**(0.11)**	**(47.47)**	**(0.00***)**	**0.02**	**0.00**	**92.8**
		**Valine_2TMS**	**2.9**	**0.30**	**0.24**	**1.25**	**0.21**			
		**3-Hydroxyisobutyrate**	**2.9**	**-0.62**	**0.21**	**-2.95**	**0.00****			
		**BMI**	**1.1**	**-0.22**	**0.12**	**-1.77**	**0.08**			
		**Sex**	**1.2**	**-0.35**	**0.13**	**-2.74**	**0.01****			

		**3-Phenyllactic acid**	**2.1**	**0.33**	**0.18**	**1.82**	**0.07**			
**High-fat non-fermented dairy**										
Unadjusted[a]	GC-MS (P)	**(Int)**	**NA**	**(3.64)**	**(0.06)**	**(61.75)**	**(0.00***)**	**0.40**	**0.16**	**27.4**
		**C17:0**	**1.4**	**0.38**	**0.17**	**2.30**	**0.02***			
		**Isoleucine**	**4.4**	**-0.98**	**0.28**	**-3.47**	**0.00*****			
		**Leucine**	**3.6**	**0.65**	**0.33**	**1.99**	**0.05***			
Adjusted[a]	GC-MS (P)	**(Int)**	**NA**	**(3.64)**	**(0.06)**	**(61.75)**	**(0.00***)**	**0.40**	**0.16**	**27.4**
		**C17:0**	**1.4**	**0.38**	**0.17**	**2.30**	**0.02***			
		**Isoleucine**	**4.4**	**-0.98**	**0.28**	**-3.47**	**0.00*****			
		**Leucine**	**3.6**	**0.65**	**0.33**	**1.99**	**0.05***			
**Low-fat non-fermented dairy**										
Unadjusted[a]	GC-MS (P)	(Int)	NA	(5.01)	(0.07)	(73.72)	(0.00***)	-0.03	0.00	99.5
		3-Hydroxyisobutyrate	1.5	-0.27	0.18	-1.50	0.14			
		Galactonate	1.5	0.12	0.11	1.07	0.29			
		Proline	1.7	0.04	0.11	0.41	0.68			
Adjusted[a]	GC-MS (P)	**(Int)**	**NA**	**(5.33)**	**(0.13)**	**(41.72)**	**(0.00***)**	**0.11**	**0.01**	**96.1**
		**Valine**	**3.0**	**0.44**	**0.27**	**1.64**	**0.10**			
		**3-Hydroxyisobutyrate**	**2.9**	**-0.69**	**0.23**	**-2.95**	**0.00****			
		**Sex**	**1.2**	**-0.37**	**0.14**	**-2.63**	**0.01****			
		**BMI**	**1.1**	**-0.24**	**0.14**	**-1.75**	**0.08**			
		**3-Phenyllactic acid**	**2.1**	**0.37**	**0.20**	**1.82**	**0.07**			

BMI, body mass index; C15:0, pentadecanoic acid; C17:0, heptadecanoic acid; FIB, food intake biomarker; GC-MS, gas chromatography mass spectrometry; MAE, mean absolute error; NA, not applicable; P, plasma; aAIC, quasi-Akaike Information Criterion; SE, standard error; VIF, variance inflation factor. Significant results are bolded: *$p \leq 0.05$, ** $p \leq 0.01$, *** $p \leq 0.001$.

[a] Intercept (Int) values for the models are provided in brackets.

metabolites MDPI

Table S9. Classification of dairy foods in the NQplus food frequency questionnaire.

Dairy Food Item	Fermentation Status	Subgroup	Fat Content[a] (g/100g food)	Fat Classification[b]
Buttermilk	Fermented	Buttermilk	0.2	Skim
Low-fat cheese (20+/30+)	Fermented	Cheese	14.1	Semi-skim
Regular cheese (40+)	Fermented	Cheese	23.9	Semi-skim
Regular cheese (48+)	Fermented	Cheese	30.3	Full fat
Cheese as snack	Fermented	Cheese	28.9	Semi-skim
Cheese with hot meal	Fermented	Cheese	28.9	Semi-skim
Fat luxury cheese	Fermented	Cheese	35.1	Full fat
Less-fat luxury cheese	Fermented	Cheese	22.0	Semi-skim
Unknown cheese	Fermented	Cheese	28.5	Semi-skim
(Fruit) quark with breakfast	Fermented	Quark	2.5	Skim
Full (fruit) yoghurt	Fermented	Yoghurt	2.8	Full fat
Full yoghurt	Fermented	Yoghurt	2.8	Full fat
Semi-skim (fruit) yoghurt	Fermented	Yoghurt	1.5	Semi-skim
Semi-skim yoghurt	Fermented	Yoghurt	1.8	Semi-skim
Skim (fruit) yoghurt	Fermented	Yoghurt	0.2	Skim
Skim yoghurt	Fermented	Yoghurt	0.2	Skim
Unknown yoghurt	Fermented	Yoghurt	1.8	Semi-skim
Butter	Non-fermented	Butter	81.1	Full fat
Skim Butter	Non-fermented	Butter	37.0	Semi-skim
Coffee cream	Non-fermented	Cream	9.4	Full fat
Cream with hot meal	Non-fermented	Cream	34.2	Full fat
Whipped cream	Non-fermented	Cream	14.8	Full fat
Milk-based ice cream	Non-fermented	Ice cream	12.0	Full fat
Diet coffee milk	Non-fermented	Milk	4.2	Full fat
Full chocolate milk	Non-fermented	Milk	2.8	Full fat
Full milk	Non-fermented	Milk	3.5	Full fat
Full-fat milk with breakfast	Non-fermented	Milk	3.5	Full fat
Regular full milk	Non-fermented	Milk	3.5	Full fat
Regular semi-skim milk	Non-fermented	Milk	1.5	Semi-skim
Semi-skim chocolate milk	Non-fermented	Milk	1.4	Semi-skim
Semi-skim coffee milk	Non-fermented	Milk	4.1	Full fat
Semi-skim milk	Non-fermented	Milk	1.5	Semi-skim
Semi-skim milk with breakfast	Non-fermented	Milk	1.5	Semi-skim
Skim chocolate milk	Non-fermented	Milk	0.5	Skim
Skim milk	Non-fermented	Milk	0.1	Skim
Unknown chocolate milk	Non-fermented	Milk	1.0	Semi-skim
Unknown coffee milk	Non-fermented	Milk	7.3	Full fat
Unknown milk	Non-fermented	Milk	1.4	Semi-skim
Milk powder for coffee	Non-fermented	Milk, powder	32.3	Full fat

[a] The fat content (g/100g) for all dairy products was determined based on values reported in the Dutch Food Composition Table [1].

[b] Fat classification was based on the guidelines set by the Dutch Dairy Commodities Act (Overheid.nl. Warenwetbesluit Zuivel), where full-fat dairy included milk and milk products with a fat content >1.80%, cheeses with a fat content ≥50%, and curd cheese/quark and cream cheese with a fat content ≥35%, semi-skim dairy included milk and milk products with a fat content ≥1.50% to ≤1.80%, cheeses with a fat content >10% to <50%, and curd cheese/quark and cream cheese with a fat content ≥10% to ≤34%, and skim dairy included milk and milk products with a fat content ≤0.5%, cheeses with a fat content ≤10%, and curd cheese/quark and cream cheese with a fat content <10%. Additional qualifiers for determining the fat content of Dutch cheeses (based on fat content dry matter) included: full-fat cheese (45+ to 60+), semi-skim cheese (10+ to 40+), and skim cheese (≤10).

Table S10. List of suppliers of analytical standards.

Compound	Supplier
3-Hydroxyisobutyrate	Sigma-Aldrich, Switzerland, ≥96.0% (sodium salt)
L-(−)-3-Phenyllactic acid	Sigma-Aldrich, Switzerland, ≥99.0%
L-Alanine	MSML kit (IROA Technologies, LLC, Bolton, MA; Gainesville, FL)
Allantoin	MSML kit (IROA Technologies, LLC, Bolton, MA; Gainesville, FL)
Alpha-aminoadipate	MSML kit (IROA Technologies, LLC, Bolton, MA; Gainesville, FL)
L-Asparagine	MSML kit (IROA Technologies, LLC, Bolton, MA; Gainesville, FL)
Blood group H disaccharide	Carbosynth, Compton, Newbury, UK
Citrulline	MSML kit (IROA Technologies, LLC, Bolton, MA; Gainesville, FL)
Delta-Gluconolactone	MSML kit (IROA Technologies, LLC, Bolton, MA; Gainesville, FL)
Galactitol	MSML kit (IROA Technologies, LLC, Bolton, MA; Gainesville, FL)
Galactonate	Sigma-Aldrich, Switzerland, ≥98.0%
Galactono-1,5-lactone	Sigma-Aldrich, Switzerland, ≥99.0% (forms 3 derivatives in solution)
D-Galactose	MSML kit (IROA Technologies, LLC, Bolton, MA; Gainesville, FL)
Gluconic acid	MSML kit (IROA Technologies, LLC, Bolton, MA; Gainesville, FL)
L-Glutamic acid	MSML kit (IROA Technologies, LLC, Bolton, MA; Gainesville, FL)
Heptadecanoic acid (C17:0)	MSML kit (IROA Technologies, LLC, Bolton, MA; Gainesville, FL)
Hippurate	MSML kit (IROA Technologies, LLC, Bolton, MA; Gainesville, FL)
Indole-3-acetaldehyde	MSML kit (IROA Technologies, LLC, Bolton, MA; Gainesville, FL)
Indole-3-acetic acid	Sigma-Aldrich, Switzerland, ≥98.0%
Indole-3-lactic acid	Sigma-Aldrich Chemie GmbH (Buchs, Switzerland)
Indole-3-propionic acid	Sigma-Aldrich Chemie GmbH (Buchs, Switzerland)
D-Lactose	MSML kit (IROA Technologies, LLC, Bolton, MA; Gainesville, FL)
Leucine	MSML kit (IROA Technologies, LLC, Bolton, MA; Gainesville, FL)
Lewis A trisaccharide	Carbosynth, Compton, Newbury, UK
L-Lysine	MSML kit (IROA Technologies, LLC, Bolton, MA; Gainesville, FL)
L-Methionine	MSML kit (IROA Technologies, LLC, Bolton, MA; Gainesville, FL)
Pentadecanoic acid (C15:0)	Sigma-Aldrich, Switzerland, ≥99.0%
L-Phenylalanine	MSML kit (IROA Technologies, LLC, Bolton, MA; Gainesville, FL)
Phenylalanyl-proline	Synpeptide Co Ltd, Shanghai, China
L-Proline	MSML kit (IROA Technologies, LLC, Bolton, MA; Gainesville, FL)
Pyroglutamate	Synpeptide Co Ltd, Shanghai, China
Taurine	MSML kit (IROA Technologies, LLC, Bolton, MA; Gainesville, FL)
L-Threonine	MSML kit (IROA Technologies, LLC, Bolton, MA; Gainesville, FL)
L-Tryptophan	MSML kit (IROA Technologies, LLC, Bolton, MA; Gainesville, FL)
L-Tyrosine	KitMSML kit (IROA Technologies, LLC, Bolton, MA; Gainesville, FL)
Valine+isoleucine	Synpeptide Co Ltd, Shanghai, China
Valyl-threonine	Synpeptide Co Ltd, Shanghai, China

Table S11. Identification features of compounds analyzed by LC-MS.

Identification	Biosample	RT (min)	m/z	Adducts	Measured neutral mass (Da)	Theoretical neutral mass (Da)
Phenylalanine	Plasma	3.23	166.0858	M+H, 2M+H	165.0788	165.07898
Tyrosine	Plasma	2.08	182.0804	M+H	181.0734	181.07389
Tryptophan	Plasma	4.07	205.0968	M+H, M+Na, 2M+Na, 2M+H	204.0898	204.08988
Indole-3-propionic acid	Plasma	8.03	190.0865	M+H, M+Na, M+H-H2O	-	189.07898
Indole-3-acetic acid	Plasma	7.1	176.0698	M+H	175.0628	175.06333
Blood group H disac-charide	Plasma	0.92	349.1113	M+Na	-	326.1213
Lewis A	Plasma	7.3	512.2018	M+H-H2O	-	529.20067
Phenylalanyl-proline	Plasma	4.94	263.1386	M+H	-	262.13174
Indole-3-lactic acid	Plasma	6.37	206.0811	M+H, M+Na	205.0741	205.07389
Proline	Plasma	1.04	116.0704	M+H	115.0634	115.06333
Lysine	Plasma	0.81	147.1125	M+H	146.1055	146.10553
Threonine	Plasma	0.93	164.029	M+2Na-H	-	119.05824
Indole-3-acetaldehyde	Plasma	4.07	160.0754	M+H	-	159.06841
Galactonate	Urine	0.97	219.048069	M+Na	196.0583	196.0583
Blood group H disac-charide	Urine	1.19	349.1101	M+Na, M+H	-	326.1213
Phenylalanyl-proline	Urine	4.98	263.1383	M+H	-	262.13174
Indole-3-lactic acid	Urine	6.4	206.081	M+H	205.074	205.07389
Proline	Urine	1.54	116.0704	M+H	-	115.06333

RT, retention time.

Table S12. Identification features of compounds analyzed by GC-MS.

Compound	Bio-sample	RT (min)	Quantifier Ion	Qualifier Ion	Ratio (Quant/Qual)	RSD QC	RI sample	RI reference
3-Hydroxyisobutyrate 2TMS	Plasma	16.71	177	218	50	40.8	1150	1151
3-Phenyllactic acid 2TMS	Plasma	26.48	193	267	14	35.9	1579	1580
Galactitol 6TMS	Plasma	30.83	307	319	120	40.5	1921	1929
Galactonate 6TMS	Plasma	31.4	292	319/333	65/35	69.0	1976	1981
Galactose 5TMS 1MEOXb	Plasma	30.58	319	160	40	23.0	1898	1898
Glutamic acid 3TMS	Plasma	26.9	246	348	8	62.8	1606	1604
Heptadecanoic acid 1TMS	Plasma	32.95	327	342	13	33.3	2136	2138
Isoleucine 2TMS	Plasma	20.5	158	218	22	44.6	1285	1285
Lactose 8TMS 1MEOXa	Plasma	38.01	361	319	50	435.3	2660	2671
Leucine 2TMS	Plasma	19.89	158	232	4.5	28.1	1263	1264
Methionine 2TMS	Plasma	25.4	176	293	10	51.5	1513	1514
Pentadecanoic acid 1TMS	Plasma	31.02	299	314	8	34.7	1939	1942
Proline 2TMS	Plasma	20.72	142	216	5	79.7	1293	1289
Valine 2TMS	Plasma	18.33	144	218	20	44.4	1207	1209
3-Phenyllactic acid 2TMS	Urine	26.44	193	267	14	29.9	1580	1580
Alanine 3TMS	Urine	22.19	188	262	17	53.2	1356	1356
Galactitol 6TMS	Urine	30.79	307	319	175	20.9	1922	1929
Galactonate 6TMS	Urine	31.36	292	319/333	65/35	57.5	1977	1981
Galactose 5TMS 1MEOXb	Urine	30.53	319	160	30	17.5	1898	1898
Hippurate 1TMS	Urine	29.99	105	236	20	18.6	1850	1845
Lactose 8TMS 1MEOXa	Urine	37.95	361	319	50	25.4	2662	2671
Pyroglutamate 2TMS	Urine	25.43	156	258	12	26.5	1518	1518

QC, quality control; RI, Kovats retention index; RT, retention time; RSD, relative standard deviation; TMS, trimethylsilyl.

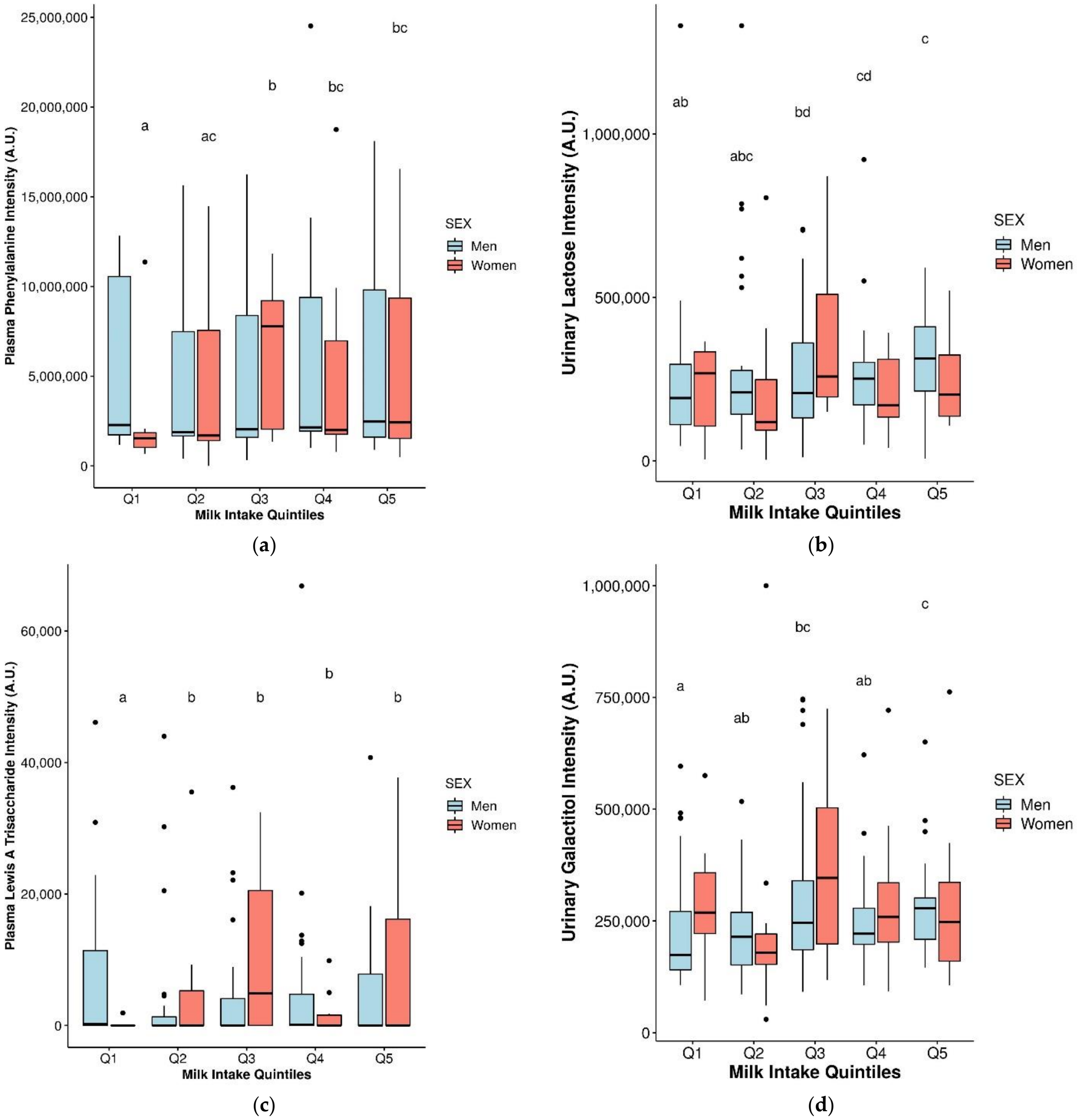

Figure S1. Differences in metabolite levels by sex-specific quintiles of milk intake: (**a**) plasma phenylalanine, (**b**) urinary lactose, (**c**) plasma Lewis A trisaccharide, and (**d**) urinary galactitol. Significance denoted by different letters ($p \leq 0.05$).

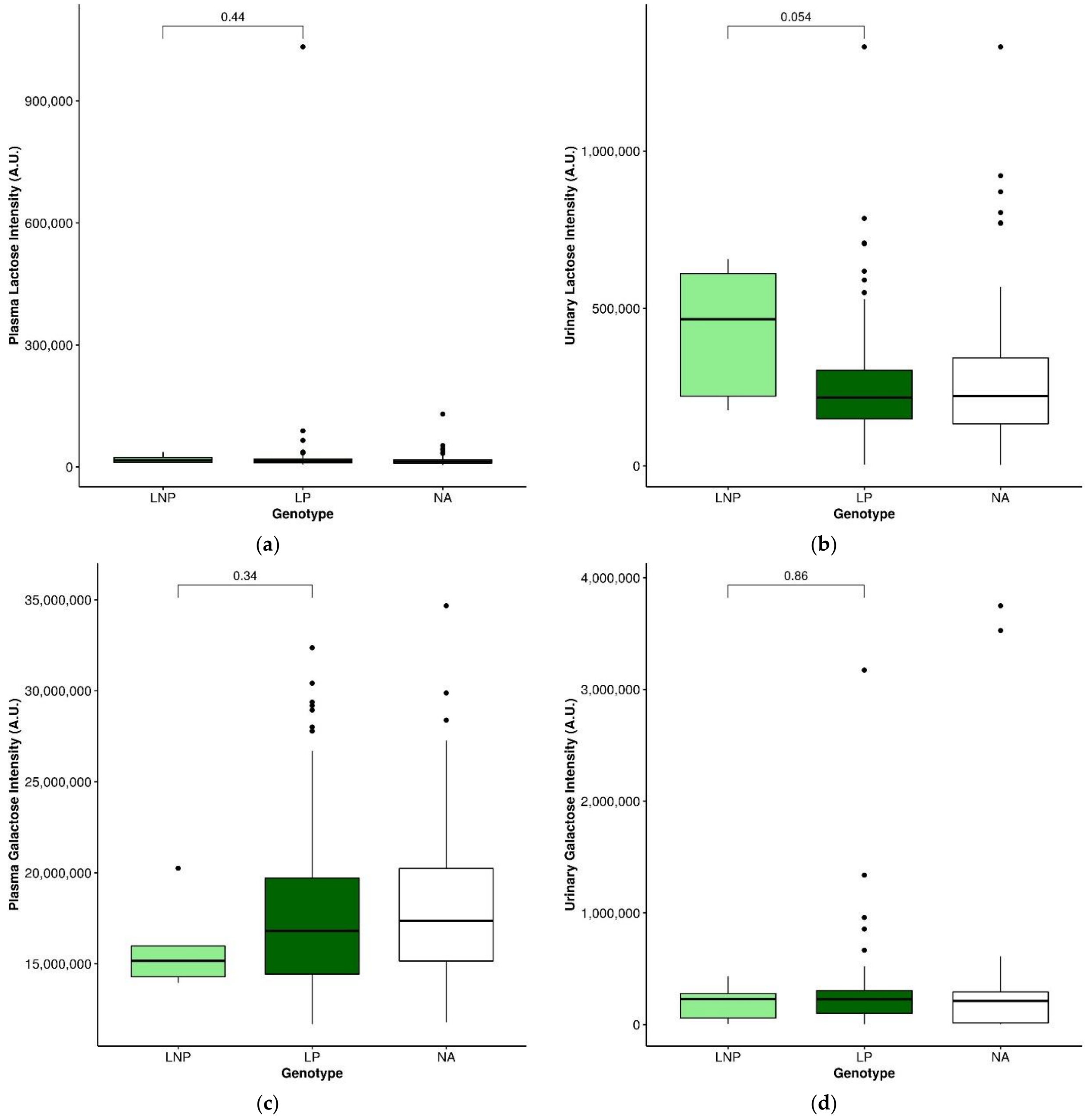
0.44
900,000
600,000
300,000
0
Plasma Lactose Intensity (A.U.)
LNP
LP
NA
Genotype
(a)
0.054
1,000,000
500,000
0
Urinary Lactose Intensity (A.U.)
LNP
LP
NA
Genotype
(b)
0.34
35,000,000
30,000,000
25,000,000
20,000,000
15,000,000
Plasma Galactose Intensity (A.U.)
LNP
LP
NA
Genotype
(c)
0.86
4,000,000
3,000,000
2,000,000
1,000,000
0
Urinary Galactose Intensity (A.U.)
LNP
LP
NA
Genotype
(d)

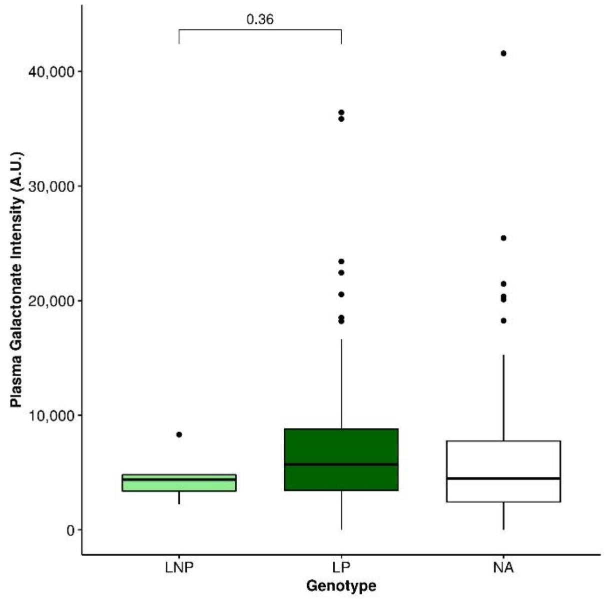

(**e**)

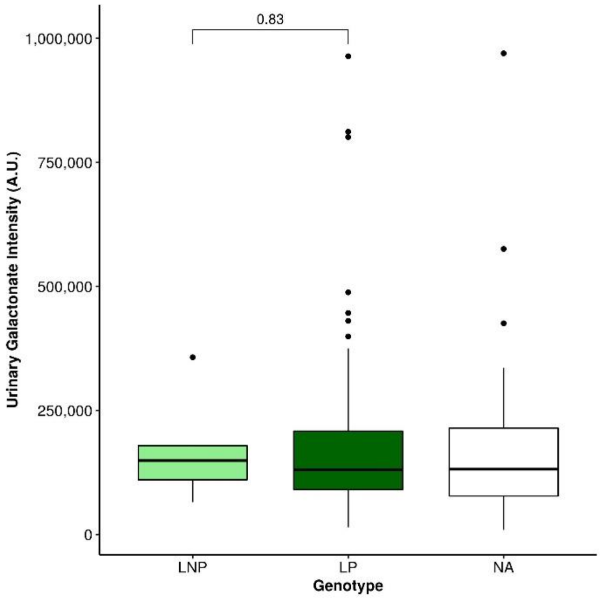

(**f**)

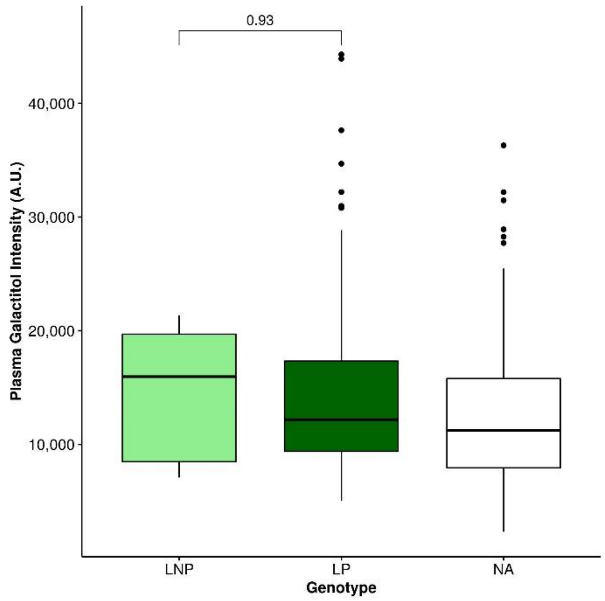

(**g**)

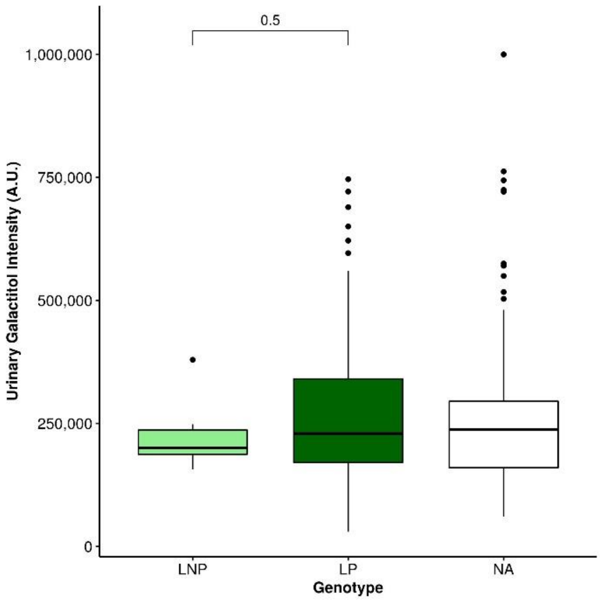

(**h**)

Figure S2. Levels of lactose metabolites. (**a**) plasma lactose (**b**) urinary lactose, (**c**) plasma galactose, (**d**) urinary galactose, (**e**) plasma galactonate, (**f**) urinary galactonate, (**g**) plasma galactitol, (**h**) urinary galactitol. Galactono-1,5-lactone was not detected in plasma or urine. LNP, lactase non-persistent; LP, lactase persistent; NA, genotype data not available.

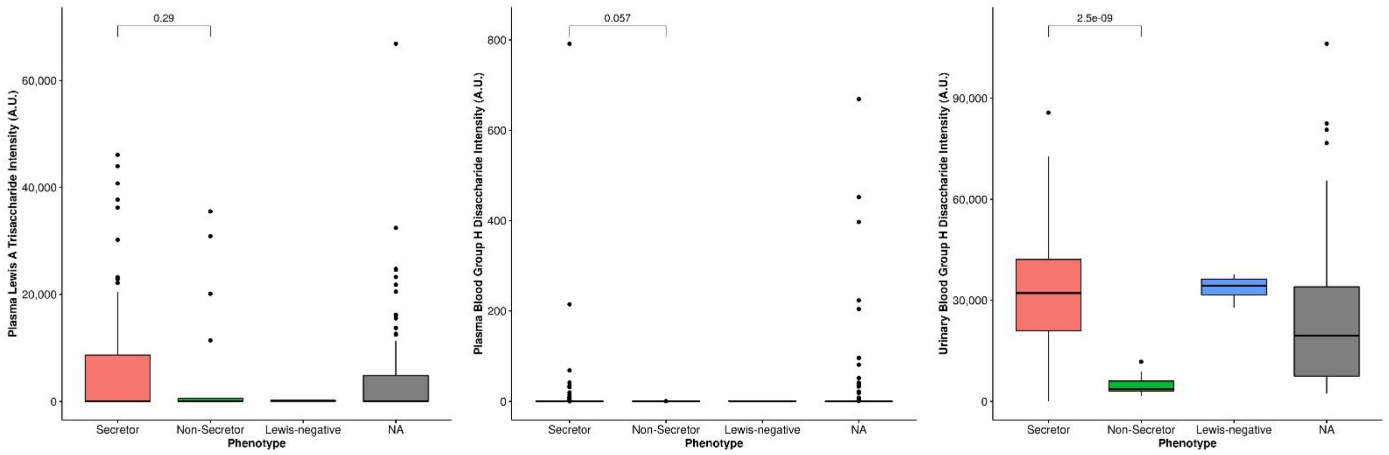

(**a**) (**b**) (**c**)

Figure S3. Levels of Lewis system-related oligosaccharides by secretion status. (**a**) plasma Lewis A trisaccharide, (**b**) plasma Blood Group H disaccharide, (**c**) urinary Blood Group H disaccharide. NA, genotype data not available.

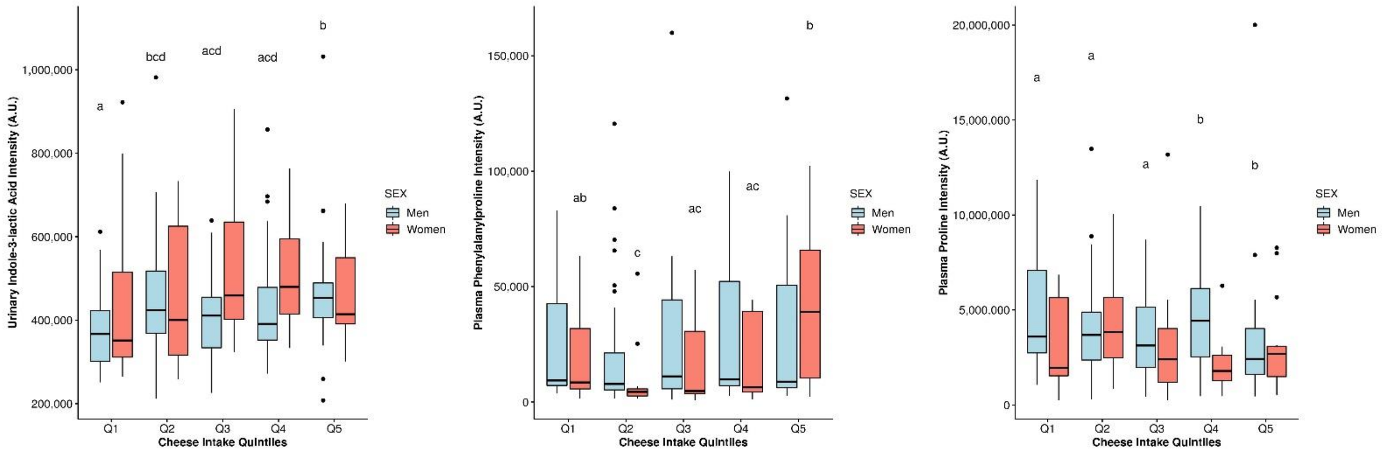

(**a**) (**b**) (**c**)

Figure S4. Differences in metabolite levels by sex-specific quintiles of cheese intake: (**a**) urinary indole-3-lactic acid, (**b**) plasma phenylalanyl-proline, (**c**) plasma proline. Significance denoted by different letters ($p \leq 0.05$).

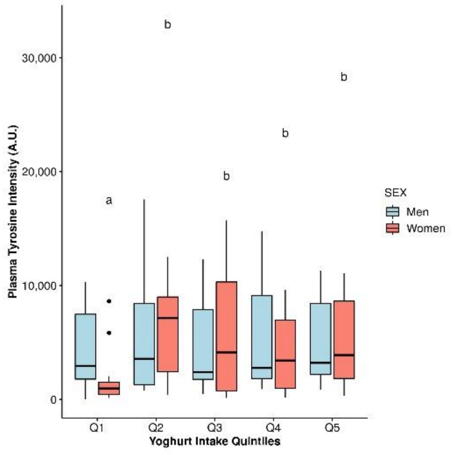

Figure S5. Differences in plasma levels of tyrosine by sex-specific quintiles of yoghurt intake. Significance denoted by different letters ($p \leq 0.05$).

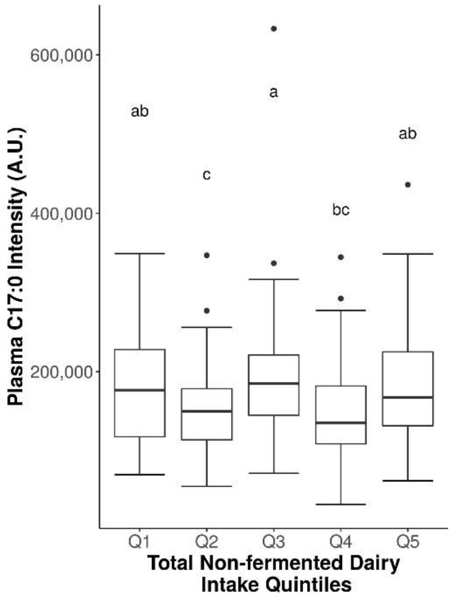

Figure S6. Differences in plasma levels of heptadecanoic acid by quintiles of total non-fermented dairy intake. Significance denoted by different letters ($p \leq 0.05$).

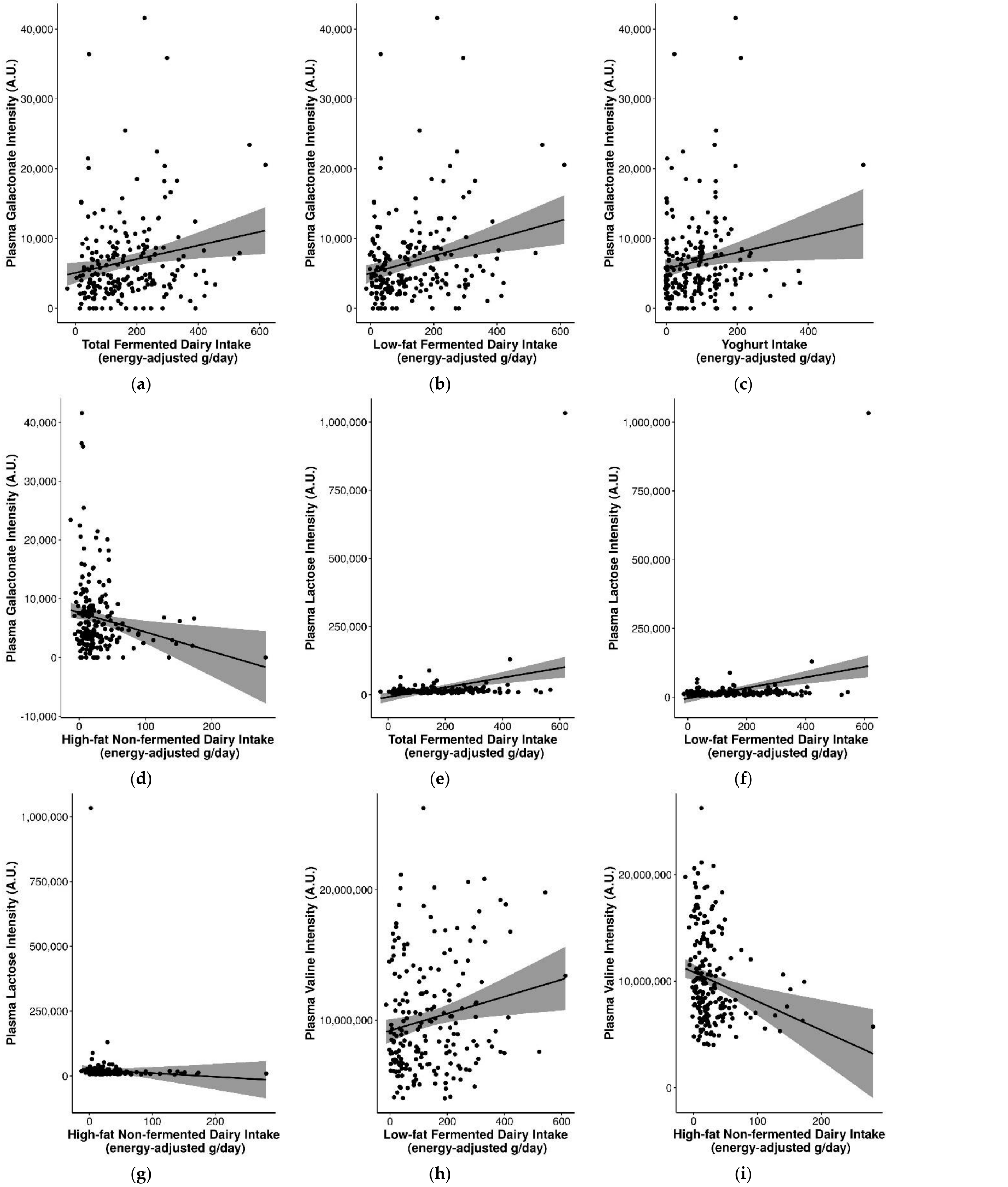

Figure S7. Plasma FIBs positively correlated with fermented dairy intake and negatively correlated with non-fermented dairy intake. Plasma galactonate significantly increasing with (**a**) total fermented dairy, (**b**) low-fat fermented dairy, and (**c**) yoghurt intake, and decreasing with (**d**) high-fat non-fermented dairy intake. Plasma lactose significantly increasing with (**e**) total fermented dairy and (**f**) low-fat fermented dairy intake, and decreasing with (**g**) high-fat non-fermented dairy intake. Plasma valine significantly increasing with (**h**) low-fat fermented dairy intake, and decreasing with (i) high-fat non-fermented dairy intake.

Supplemental References

1. The Dutch National Institute for Public Health and the Environment (RIVM) Nevo-Tabel. Nederlands Voedingsstoffenbestand. Den Haag: Voedingscentrum; **2011**. Available online: https://nevo-online.rivm.nl/ (accessed on 8 December 2020).
2. Trimigno, A.; Münger, L.; Picone, G.; Freiburghaus, C.; Pimentel, G.; Vionnet, N.; Pralong, F.; Capozzi, F.; et al. GC-MS Based Metabolomics and NMR Spectroscopy Investigation of Food Intake Biomarkers for Milk and Cheese in Serum of Healthy Humans. *Metabolites* **2018,** *8* (2), 26. DOI: 10.3390/metabo8020026.
3. Pimentel, G.; Burton, K.J.; von Ah, U.; Butikofer, U.; Pralong, F.P.; Vionnet, N.; Portmann, R.; Vergères, G. Metabolic Footprinting of Fermented Milk Consumption in Serum of Healthy Men. *J. Nutr.* **2018,** *148* (6), 851-860. DOI: 10.1093/jn/nxy053.
4. Münger , L.H.; Trimigno, A.; Picone, G.; Freiburghaus, C.; Pimentel, G.; Burton, K.J.; Pralong, F.P.; Vionnet, N.; Capozzi, F.; Badertscher, R.; et al. Identification of Urinary Food Intake Biomarkers for Milk, Cheese, and Soy-Based Drink by Untargeted GC-MS and NMR in Healthy Humans. *J. Proteome Res.* **2017,** *16* (9), 3321-3335. DOI: 10.1021/acs.jproteome.7b00319.
5. Pimentel, G.; Burnand, D.; Munger, L.H.; Pralong, F.P.; Vionnet, N.; Portmann, R.; Vergères, G. Identification of Milk and Cheese Intake Biomarkers in Healthy Adults Reveals High Interindividual Variability of Lewis System-Related Oligosaccharides. *J. Nutr.* **2020,** *150* (5), 1058-1067. DOI: 10.1093/jn/nxaa029.
